# Expedient synthesis and luminescence sensing of the inositol pyrophosphate cellular messenger 5-PP-InsP_5_†

**DOI:** 10.1039/d2sc06812e

**Published:** 2023-04-13

**Authors:** Megan L. Shipton, Fathima A. Jamion, Simon Wheeler, Andrew M. Riley, Felix Plasser, Barry V. L. Potter, Stephen J. Butler

**Affiliations:** a Department of Chemistry, Loughborough University Epinal Way, Loughborough LE11 3TU UK S.J.Butler@lboro.ac.uk; b Medicinal Chemistry & Drug Discovery, Department of Pharmacology, University of Oxford Mansfield Road Oxford OX1 3QT UK barry.potter@pharm.ox.ac.uk

## Abstract

Inositol pyrophosphates are important biomolecules associated with apoptosis, cell growth and kinase regulation, yet their exact biological roles are still emerging and probes do not exist for their selective detection. We report the first molecular probe for the selective and sensitive detection of the most abundant cellular inositol pyrophosphate 5-PP-InsP_5_, as well as an efficient new synthesis. The probe is based on a macrocyclic Eu(iii) complex bearing two quinoline arms providing a free coordination site at the Eu(iii) metal centre. Bidentate binding of the pyrophosphate group of 5-PP-InsP_5_ to the Eu(iii) ion is proposed, supported by DFT calculations, giving rise to a selective enhancement in Eu(iii) emission intensity and lifetime. We demonstrate the use of time-resolved luminescence as a bioassay tool for monitoring enzymatic processes in which 5-PP-InsP_5_ is consumed. Our probe offers a potential screening methodology to identify drug-like compounds that modulate the activity of enzymes of inositol pyrophosphate metabolism.

## Abstract 2

We report the first molecular probe for the selective detection of the most abundant cellular inositol pyrophosphate 5-PP-InsP_5_, supported by computational chemistry, as well as an efficient new synthesis.

## Graphical abstract

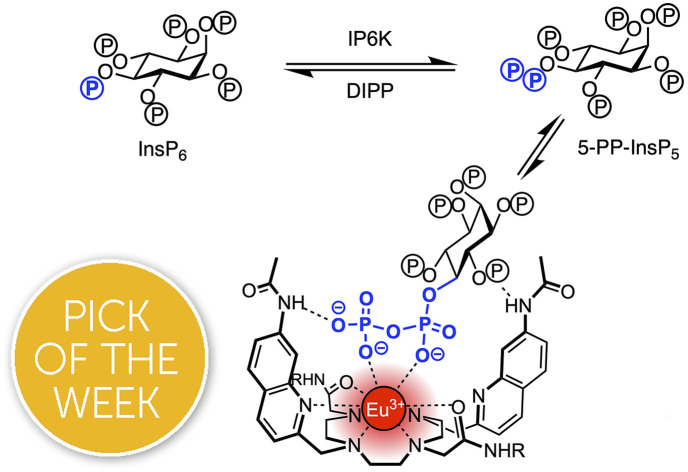

## Introduction

Inositol phosphates are highly phosphorylated small molecules of paramount importance to cellular signalling. *Myo*-inositol hexakisphosphate (InsP_6_, also called phytate) is a ubiquitous molecule in eukaryotic cells that has critical roles in signal transduction and cellular regulation. Inositol pyrophosphates (PP-InsPs), derived from inositol phosphates such as InsP_6_, possess one or more highly energetic phosphoanhydride bonds and represent the most highly phosphorylated species in nature. Because PP-InsPs are sensors and regulators of energy homeostasis in the cell they participate in a range of processes such as apoptosis, protein pyrophosphorylation, cell migration and cancer metastasis, intracellular trafficking, cell growth and kinase regulation^1,2^ with implications in disease.^3^

**Fig. 1:**
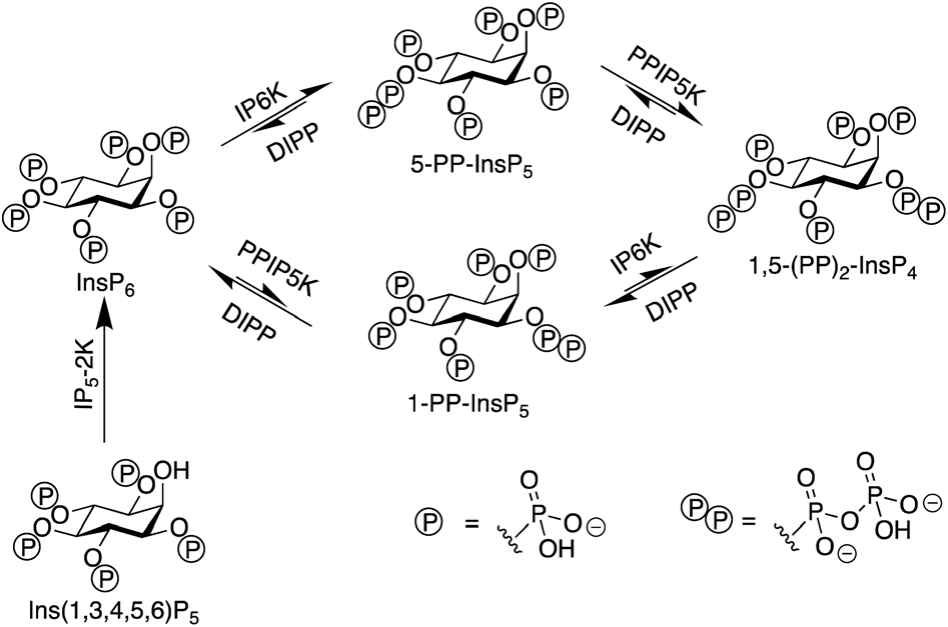
The interconversion of inositol pyrophosphates in mammals.

There are two groups of kinase enzymes in humans [InsP_6_ kinases (IP6Ks) and PP-InsP_5_ kinases (PPIP5Ks)] that can form pyrophosphate groups at either the 5-position or the 1-position of the phosphorylated inositol ring of InsP_6_.^4^ These pyrophosphate groups can be cleaved through the action of a group of promiscuous phosphatases called diphosphoinositol polyphosphate phosphohydrolases (DIPPs) (Fig. 1). The reversible interconversion between PP-InsP_5_/InsP_6_ may be considered as an ADP/ATP equivalent in protein dephosphorylation and bioenergetics,^1^ hence the development of synthetic probes that can discriminate between pyrophosphorylated inositol polyphosphates and InsP_6_ is of particular current interest.

**Scheme 1:**
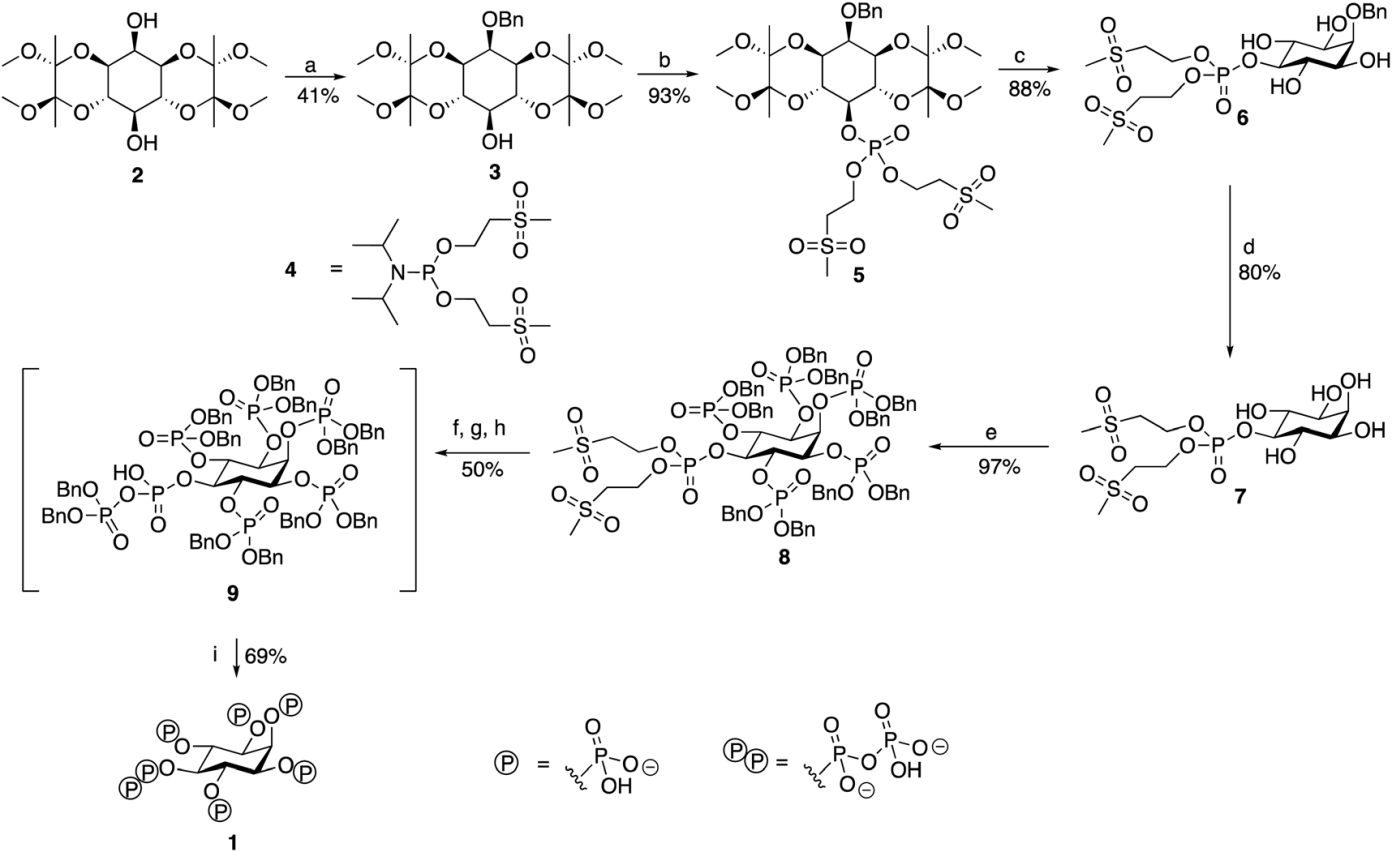
Synthesis of 5-PP-InsP_5_. Reagents and conditions: (a) DMF, NaH, BnBr; (b) (1) DCM, 5-phenyl-1*H*-tetrazole, (OMSE)_2_PN(^*i*^Pr)_2_ reagent 4, 16 h (2) *m*CPBA, −78 °C-RT; (c) TFA, H_2_O; (d) THF, CH_3_COOH, H_2_O, H_2_, Pd(OH)_2_/C; (e) (1) DCM, 5-phenyl-1*H*-tetrazole, (OBn)_2_PN(^*i*^Pr)_2_, 16 h (2) *m*CPBA, −78 °C-RT; (f) DBU, BSTFA; (g) MeOH, TFA; (h) (1) CDCl_3_, 5-phenyl-1*H*-tetrazole, (OBn)_2_PN(^*i*^Pr)_2_, 3 h (2) *m*CPBA, −78 °C-RT; (i) MeOH, THF, 1 M TEAB, H_2_, Pd(OH)_2_/C, 72 h.

The most abundant inositol pyrophosphate in mammals is 5-diphosphoinositol pentakisphosphate, 5-PP-InsP_5_ (Fig. 1 and Scheme 1) whose intracellular concentration ranges between 0.5–5.0 μM.^1,2^ Determining the exact biological functions of 5-PP-InsP_5_ and other such pyrophosphates is an ongoing pursuit and is deemed highly challenging,^5^ particularly in view of their variable modes of action, different regioisomers and the limited chemical, and especially analytical, tools available. However, 5-PP-InsP_5_ has been identified recently as the elusive regulator of Na^+^/K^+^-ATPase.^6^ The antifungal and anti-angiogenic drug itraconazole requires 5-PP-InsP_5_ to inhibit cell motility.^7^ Additionally, the participation of the 5-PP-InsP_5_ regioisomer, rather than InsP_6_ or other inositol pyrophosphates [1-PP-InsP_5_ and 1,5-(PP)_2_-InsP_4_] in glucose homeostasis was demonstrated.^8^ 5-PP-InsP_5_ is thus a G-protein coupled receptor (GPCR) second messenger that is able to regulate synaptotagmin-7 (Syt7) dependent insulin release.^9^

As a result of such emerging important functions as above, expedient synthetic routes to generate inositol pyrophosphates and analogues,^10^ as well as tools to monitor their levels that could lead to the development of high throughput *in vitro* screening methods, are becoming more urgent and relevant. The ability to track the intracellular activity of 5-PP-InsP_5_ quantitatively would be extremely useful for developing an accurate understanding of its cellular function. Moreover, library screening assay methodology might allow identification of druglike compounds to interfere with the enzymes of inositol pyrophosphate metabolism.

Molecular receptors that elicit a luminescence response upon binding a specific guest can provide a rapid and sensitive method for quantifying target species in aqueous biological media and living cells.^11–14^ Several molecular receptors for inositol phosphates in general have been developed for *in vitro* analyses;^15–20^ however, to the best of our knowledge there are no examples of receptors selective for inositol pyrophosphates. Anslyn developed a receptor for inositol trisphosphates (InsP_3_s) based on organic scaffolds substituted with phosphate-recognition moieties such as guanidinium or imidazolium groups.^21^ More recently, Bowman-James developed a supramolecular receptor for InsP_6_, trapping the anion in host–guest sandwich structures involving two picolinamide macrocycles that bind InsP_6_*via* hydrogen bonding and electrostatic interactions.^22^

To devise a molecular probe capable of the selective and sensitive detection of 5-PP-InsP_5_ we first set out to develop an expedient new synthetic strategy of the ligand. The scalable synthesis and purification of such highly phosphorylated water-soluble ligands is generally deemed to be difficult, particularly in terms of protecting group strategies. Although routes to 5-PP-InsP_5_ have been published, these often constructed the pyrophosphate group using a P(v) reagent to attach the β-phosphate precursor, in the process generating a highly unstable tri-protected P(v)–P(v) moiety before the final deprotection step.^23,24^ We chose to employ P(iii) methodology to build the pyrophosphate, resulting in the formation of a mixed P(iii)–P(v) anhydride prior to oxidation to form a di-protected P(v)–P(v) moiety. The use of such P(iii) phosphitylating agents has been adopted in the syntheses of other regioisomers of InsP_7_ and InsP_8_,^25,26^ as well as in our own work.^27,28^ A notable feature of the route in the present study is the use of the hydrogenation-resistant methylsulfonylethyl (MSE) groups to protect the phosphate attached to the 5-position.

## Results and discussion

### Synthesis of 5-PP-InsP_5_

The route (Scheme 1, see also ESI) begins from 1,6:3,4-bis-[*O*-(2,3-dimethoxybutane-2,3-diyl)]-*myo*-inositol (2) synthesised from inositol, as described.^29^ This was benzylated regioselectively at the axial 2-position hydroxyl, before being phosphorylated at the remaining exposed hydroxyl in the 5-position. The regioselectivity of this benzylation reaction has been observed before, although the reason for its occurrence is unknown.^29^

This first phosphorylation employed a phosphoramidite to attach a phosphate precursor protected by methylsulfonylethyl (MSE) protecting groups to the 5-position. The bis[(methylsulfonyl) ethyl]diisopropylphosphoramidite (4) required was synthesised in 49% yield using a version of the method described.^30^ Following oxidation with *m*CPBA, the protected monophosphate product (5) was collected in 93% yield. The BDA-protecting groups were removed with TFA to generate tetraol (6) in 88% yield. The benzyl group attached to the 2-position was cleaved *via* hydrogenation, and the product was lyophilised to generate pentaol (7) in 80% yield. The use of MSE-protecting groups was crucial during this step as they are resistant to hydrogenation, thereby enabling the easy selective removal of only the benzyl group attached to the 2-position. Phosphorylation using dibenzyl phosphoramidite then allowed benzyl-protected phosphate groups to be attached to the 1, 2, 3, 4 and 6-positions of the inositol ring. An excess of phosphoramidite and 5-phenyl-1*H*-tetrazole activator was required, but the phosphorylation was completed to generate the protected hexakisphosphate (8) in 97% yield. Use of a protecting group such as benzyl for the phosphates attached to these positions was necessary as it is an orthogonally-stable protecting group to the MSE groups attached to the phosphate at the 5-position. This enabled selective deprotection of the 5-position phosphate in the subsequent step to begin the construction of the pyrophosphate moiety only at this position.

The next stage was a multi-step sequence to construct the pyrophosphate moiety. These steps had to be performed in quick succession as some of the intermediates proved to be unstable. The first step was the simultaneous cleavage of the base-labile MSE groups and silylation of the newly exposed phosphate with DBU and BSTFA. The next was removal of the silyl groups with methanol and TFA, the progress of which was confirmed using ^31^P NMR spectroscopy. After methanolysis, the product was dried thoroughly on a vacuum line. Phosphorylation of the deprotected 5-position phosphate was achieved using benzyl-protected phosphoramidite and 5-phenyl-1*H*-tetrazole to generate the mixed P(iii)–P(v) anhydride that was subsequently oxidised using *m*CPBA to form the protected pyrophosphate (9). Importantly, an excess of 5-phenyl-1*H*-tetrazole was required during this phosphorylation to suppress competing cyclisation reactions. Following oxidation, the traditional aqueous work-up for phosphorylations was not carried out due to the instability of the protected pyrophosphate 9, although 9 was sufficiently stable to survive purification *via* flash chromatography with a silica column that had been deactivated with triethylamine immediately before the final deprotection step. The benzyl groups of the protected pyrophosphate precursor were removed in a final hydrogenation step using Pearlman's catalyst and in the presence of TEAB buffer to provide a source of counterions. Addition of chelating agent EDTA was needed to obtain sharp signals in the initial ^31^P NMR spectrum of the crude product. The product 5-PP-InsP_5_ was then purified through reverse-phase ion pair chromatography (0–10% MeCN in 0.05 M TEAB), with the phosphate-containing product fractions identified using a Briggs phosphate assay. The purified triethylammonium salt of 5-PP-InsP_5_ (1, 35% isolated yield for the entire pyrophosphate formation sequence from compound 8) was lyophilised to a colourless glass and was stored at −20 °C. It was more stable than initially expected, showing no signs of degradation by either ^1^H NMR or ^31^P NMR spectroscopy when left at room temperature for over a month.

### Molecular probe for 5-PP-InsP_5_

**Fig. 2:**
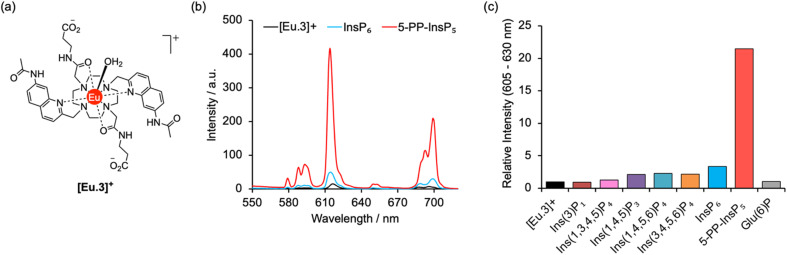
Selective increase in emission of [Eu.3]^+^ for 5-PP-InsP_5_. (a) Molecular structure of [Eu.3]^+^ (b) change in europium emission spectra of [Eu.3]^+^ (5 μM) in the presence of 250 μM 5-PP-InsP_5_ (red line) and InsP_6_ (blue line). (c) Large enhancement in intensity of the Δ*J* = 2 emission band (605–630 nm) of [Eu.3]^+^ (5 μM) upon addition of 5-PP-InsP_5_ compared with the negligible response with a range of inositol phosphates and glucose-6-phosphate (250 μM each). Measured in 10 mM HEPES, pH 7.0, 295 K, *λ*_ex_ = 330 nm.

A molecular probe of utility should be tuned to a physiological concentration range (0.5–5.0 μM) and display minimal interference from other inositol phosphates, particularly InsP_6_. Luminescent lanthanide probes are attractive for this purpose as they offer long-lived emission enabling time-resolved measurements to remove background fluorescence from the sample, and line-like emission spectra that encode information about the local coordination environment.^31–35^ We previously developed a family of macrocyclic Eu(iii) complexes whose luminescence is enhanced upon binding the nucleoside di- and triphosphates ADP and ATP, whereas all monophosphates caused much smaller changes in emission.^36–39^ Within this family we found that [Eu.3]^+^ (Fig. 2a), bearing two quinoline arms and two neutral carbonyl amide donors, exhibits the strongest binding to ADP (log *K*_a_ = 5.7)^36,39^ in aqueous solution and was therefore considered the lead candidate for sensing of 5-PP-InsP_5_. The UV-Vis absorption spectrum of [Eu.3]^+^ in aqueous buffer (10 mM HEPES, pH 7.0, 295 K) comprises a broad band centred at 330 nm (Fig. S1†). Upon excitation at 330 nm the complex displays emission in the red region of the visible spectrum, well separated from the absorption band and featuring a pronounced Δ*J* = 2 band between 605–630 nm and two discernible components within the Δ*J* = 4 band around 675–705 nm (Fig. S1†).

The ability of complex [Eu.3]^+^ to bind and sense 5-PP-InsP_5_ was examined in 10 mM HEPES buffer at pH 7.0. Addition of 250 μM 5-PP-InsP_5_ (50 equivalents) resulted in a substantial 22-fold enhancement in emission intensity of [Eu.3]^+^ within the Δ*J* = 2 band (605–630 nm) and subtle changes in spectral shape (Fig. 2b and S2†). This is consistent with coordination of the pyrophosphate group of 5-PP-InsP_5_ to the Eu(iii) metal centre and displacement of the bound water molecule, thereby reducing a major pathway for non-radiative decay of the Ln(III) ion's electronically excited state. In contrast, adding 250 μM InsP_6_ caused only a minor 3-fold increase in emission intensity, indicating a weak interaction with this anion. Similarly, very small changes in emission were observed for a wide range of common cellular inositols bearing phosphate groups to a variable degree and the carbohydrate glucose-6-phosphate (Fig. 2c and S3†).

Emission lifetimes of [Eu.3]^+^ measured in H_2_O and D_2_O (Table 1) in the absence and presence of 5-PP-InsP_5_ revealed a reduction in the number of bound water molecules, *q*, from one to zero (within experimental error), supporting direct coordination of 5-PP-InsP_5_ to the Eu(iii) centre. In comparison, we found that InsP_6_ did not reduce the hydration state, consistent with very weak binding.

**Table 1:** Emission lifetimes, hydration state *q*, and apparent binding constants for [Eu.3]^+^ with 5-PP-InsP_5_ and InsP_6_ (measured in 10 mM HEPES at pH 7.0)

Sample	*τ*_H_2_O_ a/ms	*τ*_D_2_O_ a/ms	*q* b	log *K*_a_^c^
[Eu.3]^+^	0.560	1.223	0.86	n/a
InsP_6_	0.585	1.361	0.87	n.d.
5-PP-InsP_5_	1.000	1.205	−0.09	5.37 ± 0.50

a Mean ± standard deviation for two independent measurements.

b Values of hydration state *q* (calculation error ± 20%) were derived using modified Horrocks equation.^40^

c Mean ± standard deviation for two independent measurements. n.d. = not determined due to minor changes in emission that prevented reliable data fitting.

An apparent binding constant between [Eu.3]^+^ and 5-PP-InsP_5_ was determined by plotting the change in the intensity ratio of the Δ*J* = 2/Δ*J* = 1 emission bands (605–630/580–600 nm) as a function of guest concentration, followed by curve fitting based on a 1 : 1 binding model (Fig. S4†). We measured a log *K*_a_ of 5.37 ± 0.50, which is notably higher than that previously determined for the inorganic anion pyrophosphate (log *K*_a_ = 4.7) under the same conditions, and slightly lower than for ADP (log *K*_a_ = 5.7).^36^ The small changes in emission intensity of [Eu.3]^+^ towards InsP_6_ prevented the accurate measurement of a binding constant. The possibility of a 2 : 1 [Eu.3]^+^:5-PP-InsP_5_ complex was also considered, wherein 5-PP-InsP_5_ is sandwiched between two Eu(iii) complexes in a manner similar to that observed for InsP_6_ by Bowman-James.^22^ However, fitting of the titration data of [Eu.3]^+^ with 5-PP-InsP_5_ to a 2 : 1 binding model showed no improvement in fitting compared with the 1 : 1 binding model (difference in the covariance of fit = 2.0 and substantial errors in the binding constants were observed).§§The luminescence titration data of [Eu.3]^+^ with 5-PP-InsP_5_ was fitted to both a 1 : 1 and 2 : 1 binding model using Bindfit [https://www.supramolecular.org]. The covariance of fit (cov_fit_) allowed comparison of the quality of the curve fitting between the 1 : 1 and 2 : 1 binding models (see ESI Fig. S4†). Due to the higher number of parameters from the 2 : 1 binding model, an improvement in the cov_fit_ by a factor greater than 3 should indicate that the 2 : 1 binding model is preferential.^47^ However, compared with the 1 : 1 binding model [Eu.3]^+^ showed no improvement in fitting for the 2 : 1 binding model, with F cov_fit_ = 2.0 and much larger errors in the estimated binding constants.

As a consequence of the higher affinity of [Eu.3]^+^ for 5-PP-InsP_5_ over pyrophosphate, an approximate 2-fold greater increase in emission intensity was found with the former anion, enabling these two substrates to be readily distinguished (Fig. S5†). Moreover, it was possible to discriminate between 5-PP-InsP_5_ and the nucleoside diphosphate ADP by their unique emission spectral shapes originating from differences in coordination environment at the metal centre, despite their similar overall binding affinities and overall increases in emission. Specifically, 5-PP-InsP_5_ gives rise to a single intense peak within the Δ*J* = 2 band at 615 nm (Fig. S5†), whereas ADP induces a prominent shoulder peak at 623 nm alongside a larger band at 617 nm. Further, within the Δ*J* = 4 band, 5-PP-InsP_5_ causes a pronounced peak at 700 nm, whereas ADP produces two equally intense bands in this region at 690 nm and 703 nm.

Thus, we have identified a luminescent probe that can bind and sense 5-PP-InsP_5_ with minimal interference from a variety of molecules bearing multiple phosphate and pyrophosphate groups. It should be noted that the regioisomer 1-PP-InsP_5_ (Fig. S6†) produces a similar, albeit less pronounced, emission change to 5-PP-InsP_5_. However, to the best of our knowledge no biological role has yet been identified for 1-PP-InsP_5_ (or indeed its enantiomer 3-PP-InsP_5_) that would merit the development of a sensor for its selective detection. Additionally, ATP induces an emission enhancement^36^ that would likely interfere with the recognition of 5-PP-InsP_5_ in more complex biological environments. Importantly, we do not anticipate such interference being an issue when applying the probe to *in vitro* enzyme monitoring or high throughput screening.

### Computational analysis of host–guest binding structures and luminescence spectra

**Fig. 3:**
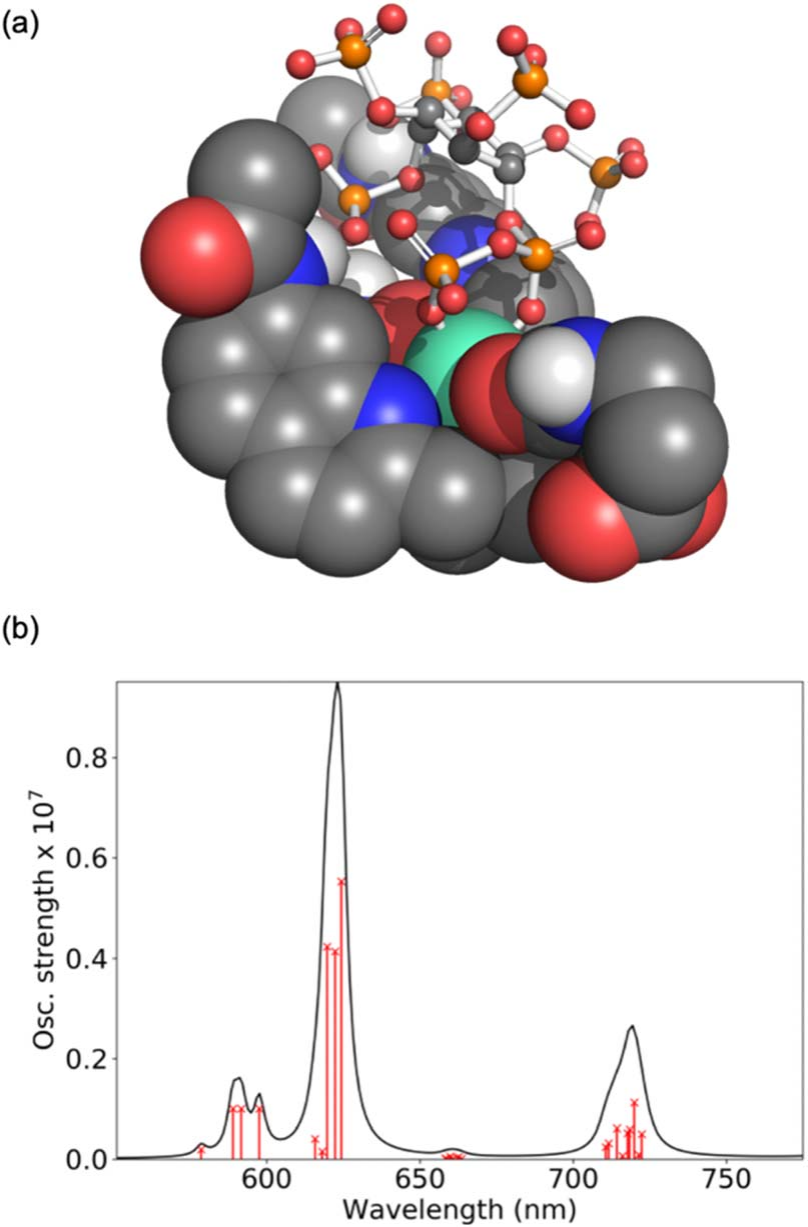
Computational analysis of 5-PP-InsP_5_ binding to [Eu.3]^+^: (a) DFT optimised geometry of the bidentate binding mode and (b) *ab initio* luminescence spectrum from ^5^D_0_ showing the individual microstates of the ^7^F term as red crosses on lines proportional to their oscillator strength.

Computations were performed to obtain further insight into host–guest binding involved in the luminescence sensing of 5-PP-InsP_5_. Structure optimizations using density functional theory (DFT)^41–43^ revealed that the most stable binding mode for 5-PP-InsP_5_ corresponds to bidentate binding of the pyrophosphate group (see Fig. 3a). One of the quinoline arms is rotated away from the Eu(iii) centre to open the second binding site needed for bidentate binding, as illustrated in Fig. S11.† The bidentate binding mode was lower in free energy by 9.2 kJ mol^−1^ compared to the second most stable binding mode featuring monodentate binding *via* the axial phosphate group, as shown in Fig. S12.† This change in free energy by 9.2 kJ mol^−1^ corresponds to a decreased binding constant by about a factor 40. This means that about 1 out of 40 bound 5-PP-InsP_5_ molecules would occupy the monodentate mode, whereas the remaining are bound in a bidentate fashion. Binding of InsP_6_ in a monodentate manner (Fig. S13†) was found to be significantly weaker than either the monodentate or the bidentate binding mode of 5-PP-InsP_5_.

To understand the enhanced binding of 5-PP-InsP_5_ relative to pyrophosphate, we have analysed the presence of hydrogen bonds within the complex. Indeed, as shown in Fig. S11,† we find three hydrogen bonds between 5-PP-InsP_5_ and the macrocyclic ligand. Two of these involve the axial phosphate (2P) group binding to amide hydrogens of [Eu.3]^+^ and the third involves an equatorial phosphate (3P) group binding to the dissociated quinoline amide arm.

The simulated luminescence spectrum^44^ for PP-InsP_5_ is shown in Fig. 3b, including transitions from the ^5^D_0_ state to the 49 microstates of the ^7^F term. The individual microstates are shown as red lines while the overall convoluted spectrum is shown in black. The simulated spectrum features a Δ*J* = 1 band split into two peaks followed by a single Δ*J* = 2 peak dominating the spectrum, and finally a Δ*J* = 4 band comprising one major peak. It agrees well with the experimental spectrum (Fig. 2b) in all these aspects, further supporting the validity of the computational model.^45^ Further spectra are shown in the ESI† highlighting the increase in the Δ*J* = 2 band with PP-InsP_5_ (and InsP_6_) compared with the water-bound complex (Fig. S9†).

### Monitoring enzymatic reactions involving 5-PP-InsP_5_

**Fig. 4:**
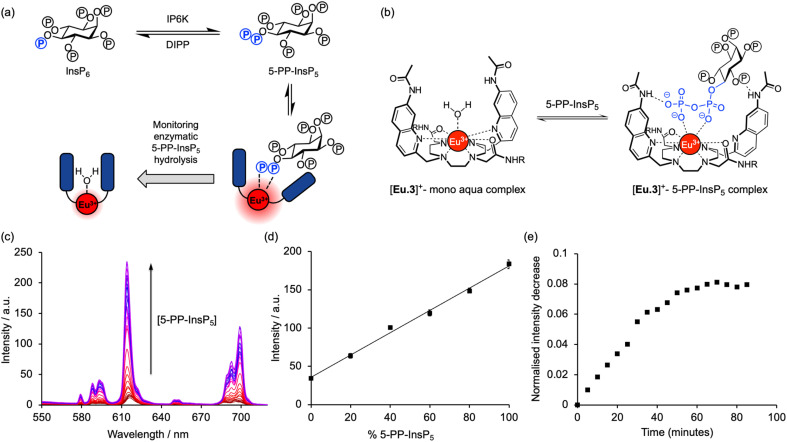
[Eu.3]^+^ can be used to monitor enzymatic hydrolysis of 5-PP-InsP_5_ to InsP_6_ by NUDT3. (a) Principle of luminescence monitoring of the reversible interconversion between PP-InsP_5_/InsP_6_ catalysed by IP6K/DIPP enzymes. [Eu.3]^+^ selectively binds 5-PP-InsP_5_ reporting its production or consumption by an increase or decrease in emission during the course of the reaction. (b) Schematic depiction of the proposed binding mode of [Eu.3]^+^ to the pyrophosphate moiety of 5-PP-InsP_5_. (c) Enhancement in Eu(iii) emission spectra upon incremental addition of 5-PP-InsP_5_ (up to 1 mM). (d) Enzyme simulation reaction demonstrating a linear increase in time-resolved emission with increasing molar ratio of 5-PP-InsP_5_/InsP_6_. Data are mean ± SEM (*r*^2^ > 0.99) from experiments performed using 5 μM [Eu.3]^+^, 100 μM total 5-PP-InsP_5_ + InsP_6_ in 10 mM HEPES, pH 7.0. (e) Representative real-time NUDT3 enzyme reaction conducted in triplicate and corrected for emission decay using identical assay mixture but omitting enzyme. Conditions: 5 μM [Eu.3]^+^, 100 μM 5-PP-InsP_5_, 1.6 μg NUDT3, 50 mM KCl, 0.05 mg mL^−1^ BSA in 40 μL of 10 mM HEPES, pH 7.0, 295 K. For all experiments: *λ*_ex_ = 292–366 nm, *λ*_em_ = 615–625 nm, integration time = 60–400 μs.

The high binding affinity of 5-PP-InsP_5_ to [Eu.3]^+^ combined with the striking increase in emission intensity and lifetime, compared with the much more subtle emission changes seen with InsP_6_ (and other inositol and carbohydrate phosphates), enabled a new luminescence method to discriminate 5-PP-InsP_5_ from InsP_6_ effectively. We exploited this to develop a microplate assay for real-time monitoring of the activity of pyrophosphatase NUDT3 (Uniprot: O95989), a DIPP phosphatase enzyme that hydrolyses the pyrophosphate group of 5-PP-InsP_5_ to generate InsP_6_ (Fig. 4a).

Initially, we confirmed the stability of the emission response of [Eu.3]^+^ with 5-PP-InsP_5_ over a 60 minutes incubation period (Fig. S6†). Next, we simulated the conversion of 5-PP-InsP_5_ into InsP_6_ (or *vice versa*) by changing the molar ratio of InsP_6_/5-PP-InsP_5_ systematically. The linear increase in time-resolved emission intensity of [Eu.3]^+^ with increasing mole fraction of 5-PP-InsP_5_ is shown in Fig. 4d. A similar linear relationship was found in the presence of bovine serum albumin, a common additive in enzyme assays (Fig. S7†). These data suggested that the luminescence signal of [Eu.3]^+^ could be correlated with the progress of a pyrophosphatase reaction.

To confirm this, we incubated the enzyme, NUDT3 with 5-PP-InsP_5_ in 10 mM HEPES buffer (containing 100 μM 5-PP-InsP_5_, 50 mM KCl, 0.05 mg mL^−1^ BSA)^46^ and were able to observe a progressive decrease in time-resolved emission intensity over 90 minutes (Fig. 4e) as NUDT3 hydrolyses 5-PP-InsP_5_ to InsP_6_. We have utilised the long-lived emission of [Eu.3]^+^ to record time-resolved emission measurements (615–625 nm, integration time = 60–400 μs), thereby removing any short-lived fluorescence arising from components in the assay sample, thus increasing signal to noise ratio.

## Conclusion

We have discovered that a coordinately unsaturated cationic lanthanide complex can be used to detect selectively the inositol pyrophosphate cellular second messenger 5-PP-InsP_5_ with low micromolar sensitivity and without interference from InsP_6_ and other common cellular inositol and carbohydrate phosphates. 5-PP-InsP_5_ was prepared *via* a new synthetic approach. The combination of selective binding of [Eu.3]^+^ to 5-PP-InsP_5_ and the resulting emission enhancement was exploited to develop a real-time assay for monitoring the enzymatic conversion 5-PP-InsP_5_ to InsP_6_. Our enzymatic assay was conducted in time-resolved format to exploit the long luminescence lifetime of [Eu.3]^+^, and thus improve signal-to-noise ratio, and in multi-well plates importantly demonstrating its potential suitability for high-throughput use. The assay is label-free and does not require expensive antibodies or chemical modification of the substrate with a fluorescent or radioactive label. Its availability should have a significant impact on the study of protein dephosphorylation and emerging cellular signalling pathways regulated by 5-PP-InsP_5_, not least those involved in glucose homeostasis.

## Data availability

ESI is available, including synthetic procedures, compound characterisation, and photophysical studies of host–guest interactions.†

## Author contributions

M. L. S.: synthesis and characterisation of compounds. F. A. J. and S. W.: europium probe studies, anion binding and sensing experiments, enzyme assay development. F. P.: computation, supervision of modelling studies, data analysis. A. M. R.: supervision and development of synthetic methods, data analysis. B. V. L. P.: conceptualisation, funding acquisition, supervision of synthetic aspects, data analysis, writing and editing of the manuscript. S. J. B.: conceptualisation, funding acquisition, supervision of europium probe studies, methodology, data analysis, writing and editing of the manuscript. All authors have contributed to, seen and approved the manuscript.

## Conflicts of interest

There are no conflicts to declare.

## Supplementary Material

SC-014-D2SC06812E-s001
