# Supplementary material for: Expedient synthesis and luminescence sensing of the inositol pyrophosphate cellular messenger 5-PP-InsP5
Source: Chem Sci. 2023 Apr 13;14(19):4979–85. doi: 10.1039/d2sc06812e (PMC10189900; doi:10.1039/d2sc06812e)
Supplement: SC-014-D2SC06812E-s001 [file SC-014-D2SC06812E-s001.pdf]

Electronic Supplementary Material (ESI) for Chemical Science.
This journal is © The Royal Society of Chemistry 2023

Supplementary Information

Expedient Synthesis and Luminescence Sensing of the Inositol Pyrophosphate Cellular Messenger 5-PP-InsP$_5$

Megan L. Shipton[b], Fathima A. Jamion[a], Simon Wheeler[a], Andrew M. Riley[b], Felix Plasser[a], Barry V. L. Potter*[b] and Stephen J. Butler*[a]

[a]*Department of Chemistry, Loughborough University, Epinal Way, Loughborough, LE11 3TU, UK.*

[b]*Medicinal Chemistry & Drug Discovery, Department of Pharmacology, University of Oxford, Mansfield Road, Oxford OX1 3QT, U.K.*

*Correspondence: s.j.butler@lboro.ac.uk and barry.potter@pharm.ox.ac.uk

Contents

Materials and methods S2

Synthesis of 5-diphosphoinositol pentakisphosphate (5-PP-InsP$_5$) S4

NMR spectra of compounds S10

Photophysical analysis of [**Eu.3**]$^+$ alone and with analytes S27

DFT-optimised molecular structures and *ab initio* luminescence spectra S30

References S33

Materials and methods

General synthetic considerations

Chemicals were purchased from Sigma-Aldrich, Acros, or Alfa Aesar. Anhydrous solvents were purchased from Sigma-Aldrich. TLC was performed on precoated plates (Merck Aluminum sheets silica 60 F254, art No. 5554). Chromatograms were visualised under UV light and by dipping plates into phosphomolybdic acid in EtOH, followed by heating. Flash column chromatography was performed using RediSep Rf disposable silica flash columns on an ISCO CombiFlash Rf automated flash chromatography machine. Ion-pair reverse-phase chromatography was performed on LiChroprep RP-18 (25-40 μm, Merck) using a BioLogic LP system (BioRad), eluting at 5 mL/min with a gradient of 0−10% MeCN in 0.05 M triethylammonium bicarbonate (TEAB) buffer, collecting 7 mL fractions. Fractions containing the target polyphosphate were identified using a qualitative Briggs phosphate assay (as described by Lampe *et al.*[1]).

Proton ^{1}HNMR and COSY spectra were recorded on a Bruker Avance III (400 MHz) spectrometer. Proton chemical shifts are reported in ppm (δ) relative to internal tetramethylsilane (TMS, δ 0.0 ppm) or with the solvent reference relative to TMS employed as the internal standard ($CDCl_3$, δ 7.26 ppm; CD_3OD, δ 3.31 ppm). The following abbreviations are used to describe the multiplicity of the chemical shifts: s, singlet; d, doublet; t, triplet; dd, double doublet; q, quartet; m, multiplet; br, broad. ^{13}CNMR and HSQC spectra were recorded on a Bruker Avance III (100 MHz) spectrometer with complete proton decoupling. Carbon chemical shifts are reported in ppm (δ) relative to TMS with the respective solvent resonance as the internal standard ($CDCl_3$, δ 77.0 ppm, d4-MeOH, δ 49.0 ppm). ^{31}PNMR spectra were recorded on a Bruker Avance III (162 MHz) spectrometer with complete proton decoupling (unless explicitly labelled as proton-coupled). Phosphorus chemical shifts are reported in ppm (δ) relative to an 85% H_3PO_4 external standard (H_3PO_4, δ 0.0 ppm). All NMR data were collected at 25°C. Melting points were determined using a Stanford Research Systems Optimelt MPA100 automated melting point system and are uncorrected. Mass spectra were recorded on a GC-MS (Agilent 7200). All reactions were carried out under an argon atmosphere employing oven-dried glassware, unless stated otherwise.

Luminescence experiments

Luminescence spectra were recorded on a Camlin Photonics luminescence spectrometer with FluoroSENS version 3.4.7.2024 software. Emission spectra were obtained using a 40 μL or 100 μL Hellma Analytics quartz cuvette (Art no. 111-10-K-40). Excitation light was set at 322 nm and emission recorded in the range 400 – 720 nm using an integration time of 0.5 seconds, increment of 1.0 nm, excitation slit of 0.2 nm and emission slit of 0.5 nm. Emission lifetime measurements were performed on the FluoroSENS instrument. Measurements were taken of 1 mL of 0.1 absorbance samples of Eu(III) complexes in 10 mM HEPES at pH 7.0, unless stated otherwise. Measurements were obtained by indirect excitation of the Eu(III) ion *via* the quinoline antennae using a short pulse of light at 322 nm followed by monitoring the integrated intensity of the light emitted at

615 nm, with 500 data points collected over a 10 millisecond time period. The decay curves were plotted in Origin Labs 2019 version 9.6.0.172, and fitted to the equation:

$$I = A_0 + A_1 e^{-kt}$$

where I is the intensity at time, *t*, following excitation, A_0 is the intensity when decay has ceased, A_1 is the pre-exponential factor and k is the rate constant for the depopulation of the excited state.

The hydration state, *q*, of the Eu(III) complexes was determined using the modified Horrocks equation:[2]

$$q\ (\mathrm{Eu}) = 1.2\ (1/\tau_{\mathrm{H2O}} - 1/\tau_{\mathrm{D2O}} - 0.25 - 0.075\mathrm{n})$$

where τ_{H2O} and τ_{D2O} are the emission lifetime times in water and D_2O, respectively, and n is the number of carbonyl-bound amide NH groups.

Anion binding titrations

Anion binding titrations were carried out in degassed 10 mM HEPES buffer at pH 7.0. Stock solutions of anions (e.g. hydrogen phosphate, AMP) containing Eu(III) complex (5 μM) were made up at 0.4, 4 and 40 mM anion. The appropriate anion stock solution was added incrementally to 100 μL of Eu(III) complex (5 μM) and the emission spectrum was recorded after each addition. The ratio of emission bands 605 – 630 nm/ 585 – 600 nm ($\Delta J = 2$ / $\Delta J = 1$) was plotted as a function of anion concentration. The data were analysed using a nonlinear least-squares curve fitting procedure, based on a 1:1 binding model described by the equation:

$$\mathrm{FB} = \frac{\frac{1}{\mathrm{K_a}} + [\mathrm{A}] + [\mathrm{Eu}] - \sqrt{(\frac{1}{\mathrm{K_a}} + [\mathrm{A}] + [\mathrm{Eu}])^2 + 4[\mathrm{A}][\mathrm{Eu}]}}{2[\mathrm{Eu}]}$$

where FB is the fraction bound, calculated by $(I-I_0)/(I_1-I_0)$ where I is the emission intensity at [A], I_0 is the initial emission intensity, and I_1 is the final emission intensity. [A] is the total concentration of anion in solution, [Eu] is the total concentration of Eu(III) complex, K_a is the apparent binding constant.

Computational details

Geometry optimisations were performed at the DFT level using the ωB97M-V functional along with the 6-31G* basis set.[3, 4] A large-core quasi-relativistic effective core potential (ECP)[5] was used for treating the core along with the $4f^6$ shell of Eu(III) and the associated (7s6p5d)/[5s4p3d] basis set was used for the valence electrons. All computations were performed using spin-restricted orbitals using a pseudo-singlet configuration. Solvation in water was modelled using Truhlar's SMD model.[6] These geometry optimisations were carried out in Q-Chem 5.4.[7]

Additional single-point computations were performed in Orca 5.0[8] using again the SMD solvent model and ωB97M-V functional but with the larger def2-TZVP basis set (long with the def2 ECP) and the gCP correction for basis-set superposition error.[9, 10] These computations replaced the Eu^{3+} atom with Y^{3+} (possessing a similar atomic radius) to avoid open-shell f-electrons. The free energies of solvation from these computations are reported in the main text. No further thermostatistical corrections were carried out.

Emission spectra were simulated using the *ab initio* complete active space self-consistent field (CASSCF) level of theory. These computations employed the ANO-RCC-VTZP basis set for the Eu atom, ANO-RCC-VDZP for heteroatoms, and ANO-RCC-VDZ for C/H atoms.[11] The active space consisted of the 6 f-electrons distributed over the 7 f-orbitals. CASSCF orbital optimizations were performed by averaging over 7 states (making up the full CAS(6,7) heptet configuration space). Using these orbitals, CASCI computations on quintets (15 roots), triplets (10 roots), and singlets (5 roots). Heptets, quintets, triplets, and singlets were combined in a spin-orbit state interaction procedure (SO-RASSI) to produce the final spin-orbit coupled states.[12] Emission spectra were computed with respect to the $^5D_0$ state, i.e. the 50th state in the spin-orbit expansion. Oscillator strengths for the quadropole allowed $\Delta J = 1$ states were fixed at a value of 10^{-8} whereas the oscillator strengths for all other states were computed via transition dipole moments in the length representation. To match the results closer to the experimental spectra, energies were shifted down by 0.4 eV. Reduced molecular models were used to make these computations feasible: the inositol phosphate in monodentate/bidentate binding mode was replaced with phosphate/pyrophosphate; functional groups of the macrocyclic ligand not bound to Eu were removed. Spectra are shown with a phenomenological broadening of 0.015 eV. These CASSCF calculations were carried out in OpenMolcas.[13]

The underlying computational research data (molecular structures and input/output files of Q-Chem, Orca and OpenMolcas) are provided *via* separate repository, DOI: 10.17028/rd.lboro.21666218.

Synthesis of 5-diphosphoinositol pentakisphosphate (5-PP-$InsP_5$)

2-*O*-Benzyl-1,6:3,4-bis-*O*-(2,3-dimethoxybutane-2,3-diyl)-*myo*-inositol (3)

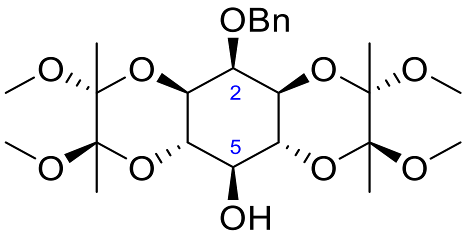

As described by Wang *et al.*,[14] sodium hydride (149 mg, 5.88 mmol, 1.2 equiv) was added over 10 minutes to a suspension of 1,6:3,4-bis-*O*-(2,3-dimethoxybutane-2,3-diyl)-*myo*-inositol (synthesised as described by Riley *et al.*,[15] 2.00 g, 4.90 mmol) in dry DMF (120 mL) under argon. After an hour of stirring at RT, benzyl bromide (0.64 mL, 5.39 mmol, 1.1 equiv) was added slowly over 30 minutes. The reaction mixture was then allowed to stir at RT for 20 h. Completion of the reaction was confirmed with TLC, before the excess sodium hydride was destroyed through the dropwise addition of MeOH (10 mL). The solution was concentrated *in vacuo* and the

residue dissolved in DCM (200 mL). The organic layer was washed with water (200 mL) and brine (200 mL), before being dried over $MgSO_4$ and concentrated. The crude product was purified by flash chromatography (P.E.-EtOAc, 0-100%) to give the pure product as a white solid (1009.9 mg, 2.03 mmol, 41% yield). **m.p.** 207.9-212.0 °C (lit.[15] 214– 216 °C); **^{1}HNMR** ($CDCl_3$, 400 Hz): 7.53-7.51 (m, 2H, Ar), 7.34-7.23 (m, 3H, Ar), 4.87 (s, 2H, C*H₂*Ar), 4.08 (t, *J*= 9.9 Hz, 2H, H-4 and H-6), 3.82 (t, *J*= 2.5 Hz, 1H, H-2), 3.67 (t, *J*= 9.5 Hz, 1H, H-5), 3.54 (dd, *J*= 2.5, 10.2 Hz, 2H, H-1 and H-3), 3.28 (s, 6H, OCH_3), 3.23 (s, 6H, OCH_3), 2.46 (s, 1H, OH), 1.32 (s, 12H, CCH_3); **^{13}CNMR** ($CDCl_3$, 100 Hz): 139.8 (Ar), 128.0 (Ar), 127.8 (Ar), 127.1 (Ar), 99.8 (*C*CH_3), 99.3 (*C*CH_3), 76.4 (C-2), 74.0 (*C*H_2Ar), 70.8 (C-5), 69.5 (C-4 and C-6), 69.3 (C-1 and C-3), 48.1 (O*C*H_3), 48.0 (O*C*H_3), 17.9 (C*C*H_3), 17.8 (C*C*H_3); **HRMS (ESI)**: *m/z* calcd $[M+Na]^+$ for $C_{25}H_{38}O_{10}$: 521.2357, found: 521.2359.

***N,N*-Diisopropylamino-bis-[2-(methylsulfonyl)ethyloxy]phosphine (4)**

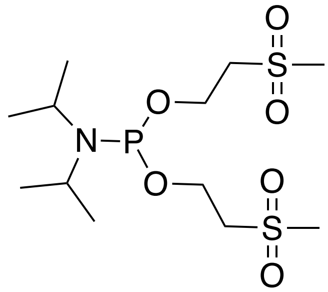

In a version of the method described by Engelsma *et al.,*[16] trichlorophosphane (0.50 mL, 5.73 mmol) was added to a solution of DIPEA (2.00 mL, 11.46 mmol, 2 equiv) in dry DCM (20 mL) under argon. This solution was then cooled to 0°C before diisopropylamine (1.63 mL, 11.46 mmol, 2 equiv) was added. The reaction mixture was allowed to stir for 10 min at 0°C. Methylsulfonylethanol (1.42 g, 11.46 mmol, 2 equiv) dissolved in dry DCM (30 mL) was then added dropwise to the reaction. The mixture was allowed to warm to RT and left to stir for 3 h. The completion of the reaction was determined by ^{31}PNMR (diagnostic product signal at 149 ppm). The reaction mixture was then diluted with EtOAc (200 mL), washed with sat. sodium bicarbonate (100 mL) and brine (100 mL). The organic layer was dried over $MgSO_4$ and concentrated *in vacuo* to yield the crude product as a yellow oil. The crude product was purified by flash chromatography (column deactivated with 1% TEA in PE prior to use, petroleum ether and EtOAc, 30 to 100%) and then recrystallized (minimum amount of DCM, approx. 2 mL, and then 150 mL diethyl ether) to give the pure product as a white, crystalline solid (1060 mg, 2.81 mmol, 49% yield). **m.p.** 73.3- 74.1 °C (lit. value[17] 75.5-77.0 °C); **^{1}HNMR** ($CDCl_3$, 400 Hz): 4.13-4.03 (m, 4H, CH_2CH_2), 3.61-3.55 [m, 2H, *C*H(CH_3)$_2$], 3.29-3.25 (m, 4H, CH_2CH_2), 3.00 (s, 6H, SCH_3), 1.20 [s, 6H, CH(CH_3)$_2$], 1.18 [s, 6H, CH(CH_3)$_2$]; **^{31}PNMR** ($CDCl_3$, 162 Hz): 149.06; **^{13}CNMR** ($CDCl_3$, 100 Hz): 57.8 (*C*H_2CH_2), 57.6 (*C*H_2CH_2), 56.4 (CH_2*C*H_2), 56.3 (CH_2*C*H_2), 43.5 [*C*H(CH_3)$_2$], 43.4 [*C*H(CH_3)$_2$], 43.0 (S*C*H_3), 24.8 [s, 6H, CH(*C*H_3)$_2$], 24.7 [s, 6H, CH(*C*H_3)$_2$]; **HRMS (ESI)**: *m/z* calcd $[M+Na]^+$ for $C_{12}H_{28}NO_6PS_2$: 400.1007, found: 400.0996.

2-*O*-Benzyl-1,6:3,4-bis-*O*-(2,3-dimethoxybutane-2,3-diyl)-*myo*-inositol-5-bis[2-(methanesulfonyl)ethyl]phosphate (5)

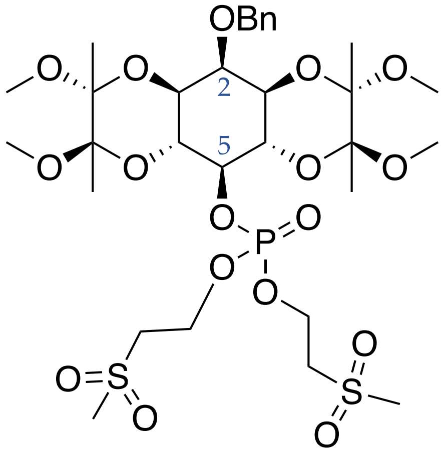

2-*O*-Benzyl-1,6:3,4-bis-*O*-(2,3-dimethoxybutane-2,3-diyl)-*myo*-inositol (**3,** 909 mg, 1.82 mmol) was dissolved in dry DCM (18 mL) and the solution put under argon. To this, 5-phenyl-1*H*-tetrazole (400 mg, 2.73 mmol, 1.5 equiv.) followed by *N,N*-diisopropylamino-bis-[2-(methylsulfonyl)ethyloxy]phosphine (**1**, 826 mg, 2.19 mmol, 1.2 equiv) were added. The reaction mixture was allowed to stir at RT for 16 h. Prior to oxidation, the reaction was analysed by ^{31}PNMR spectroscopy to confirm that phosphitylation had been successful. The reaction mixture was then cooled to -78 °C, before *m*CPBA (900 mg, 70% pure, 3.65 mmol, 2.0 equiv) was added. The solution was allowed to warm to RT and was then diluted with EtOAc (150 mL). This organic phase was washed with 10% sodium sulphite solution (3x20 mL), dried over $MgSO_4$ and concentrated *in vacuo* to yield the crude product. The product was purified by flash chromatography (EtOAc-MeOH, 0-20%) and the pure product collected as a white foam (1339 mg, 1.69 mmol, 93% yield). **^{1}HNMR** ($CDCl_3$, 400 Hz): 7.44-7.42 (m, 2H, Ar), 7.27-2.17 (m, 3H, Ar), 4.77 (s, 2H, C*H₂*Ar), 4.60-4.57 (m, 2H, MSE CH_2), 4.53-4.48 (m, 2H, MSE CH_2), 4.33 (q, *J*= 9.0 Hz, 1H, H-5), 4.11 (t, *J*= 9.8 Hz, 2H, H-4 and H-6), 3.75 (s, 1H, H-2), 3.50 (d, *J*= 10.0 Hz, 2H, H-1 and H-3), 3.45-3.39 (m, 2H, MSE CH_2), 3.30-3.26 (m, 2H, MSE CH_2), 3.19 (s, 6H, OCH_3), 3.15 (s, 6H, OCH_3), 2.94 (s, 6H, SCH_3), 1.24 (s, 6H, CCH_3), 1.20 (s, 6H, CCH_3); **^{31}PNMR** ($CDCl_3$, 162 Hz): -2.44; **^{13}CNMR** ($CDCl_3$, 100 Hz): 139.4 (Ar), 128.1 (Ar), 127.8 (Ar), 127.3 (Ar), 99.8 (*C*CH_3), 99.5 (*C*CH_3), 78.3 (C-5), 76.0 (C-2), 74.2 (*C*H_2Ar), 68.9 (C-1 and C-3), 68.1 (C-4 or C-6), 68.0 (C-4 or C-6), 61.6 (MSE CH_2), 61.5 (MSE CH_2), 55.2 (MSE CH_2), 55.1 (MSE CH_2), 48.2 (OCH_3x2), 42.8 (SCH_3), 17.79 (CCH_3), 17.75 (CCH_3); **HRMS (ESI)**: *m/z* calcd $[M+Na]^+$ for $C_{31}H_{51}O_{17}PS_2$: 813.2197, found: 813.2188.

2-*O*-Benzyl-*myo*-inositol-5-bis[2-(methanesulfonyl)ethyl]phosphate (6)

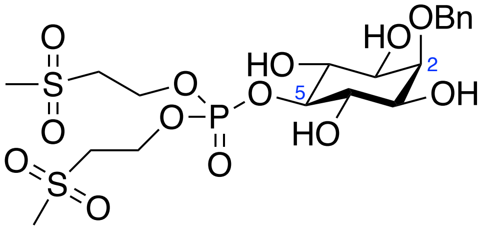

2-*O*-Benzyl-1,6:3,4-bis-*O*-(2,3-dimethoxybutane-2,3-diyl)-*myo*-inositol-5-bis[2-(methanesulfonyl)ethyl] phosphate (**5**, 396 mg, 0.50 mmol) was dissolved in a mixture of TFA (4.5 mL) and water (0.5 mL). The reaction mixture was allowed to stir for 10 min under argon, during which time the solution turned dark yellow. The solution was then concentrated *in vacuo* without heating. The resultant residue was dissolved in methanol and concentrated *in vacuo* to yield the crude product as a white solid. The product was purified by recrystallization from boiling ethanol to yield the pure product as colourless crystals (249 mg, 0.44 mmol, 88% yield). ^{1}**HNMR** (d4-MeOH, 400 Hz): 7.43- 7.41 (m, 2H, Ar), 7.33- 7.23 (m, 3H, Ar), 4.61-4.56 (m, 6H, CH_2Ar and MSE CH_2), 4.10 (q, *J*= 9.0 Hz, 1H, H-5), 3.92 (t, *J*= 2.6 Hz, 1H, H-2), 3.82 (t, *J*= 9.6 Hz, 2H, H-4 and H-6), 3.53 (t, *J*= 5.5 Hz, 4H, MSE CH_2), 3.48 (dd, *J*= 9.9, 2.5 Hz, 2H, H-1 and H-3), 3.04 (s, 6H, SCH_3); ^{31}**PNMR**: (d6-DMSO, 162 Hz) -2.26; ^{13}**CNMR** (d6-DMSO, 100 Hz): 139.8 (Ar), 128.0 (Ar), 127.1 (Ar), 83.5 (C-5), 81.8 (C-2), 74.4 (*C*H_2Ar), 71.5 (C-1 and C-3), 71.4 (C-4 and C-6), 61.11 (MSE CH_2), 61.06 (MSE CH_2), 54.2 (MSE CH_2), 54.1 (MSE CH_2), 42.0 (SCH_3); **HRMS (ESI)**: *m/z* calcd $[M+H]^+$ for $C_{19}H_{31}O_{13}PS_2$: 563.1016, found: 563.1014.

***myo*-Inositol-5-bis[2-(methanesulfonyl)ethyl]phosphate (7)**

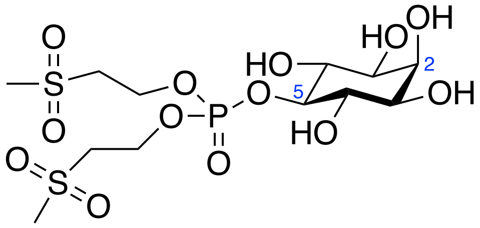

2-*O*-Benzyl-*myo*-inositol-5-bis[2-(methanesulfonyl)ethyl]phosphate (**6**, 100 mg, 0.178 mmol) was dissolved in THF (2.5 mL) and ultrapure water (0.5 mL). To this solution, three drops of acetic acid and palladium hydroxide on carbon (20 mg, 20%) were added. The reaction mixture was allowed to stir under hydrogen for 24 hours at RT. The catalyst was then filtered off, and the solution was concentrated *in vacuo*. The residue was dissolved in ultrapure water and lyophilised to yield the product as a white solid (66.9 mg, 0.142 mmol, 80% yield). Due to compound instability when in solution, the compound was only partially characterised before being used in the next step. ^{1}**HNMR** (d6-DMSO, 400 Hz): 4.98 (d, *J*= 5.7 Hz, 2H), 4.70 (d, *J*= 3.7 Hz, 1H), 4.69 (d, *J*= 5.9 Hz, 2H), 4.44-4.34 (m, 4H), 3.93 (q, *J*= 9.1 Hz, 1H), 3.71 (q, *J*= 3.0 Hz, 1H), 3.60-3.54 (m, 2H), 3.51 (t, *J*=5.7 Hz, 4H), 3.23-3.18 (m, 2H), 3.01 (s, 6H); ^{31}**PNMR** (d6-DMSO, 162 Hz): -2.21.

***myo*-Inositol-1,2,3,4,6-pentakis(dibenzyl)phosphate-5-bis[2-(methanesulfonyl)ethyl]-phosphate (8)**

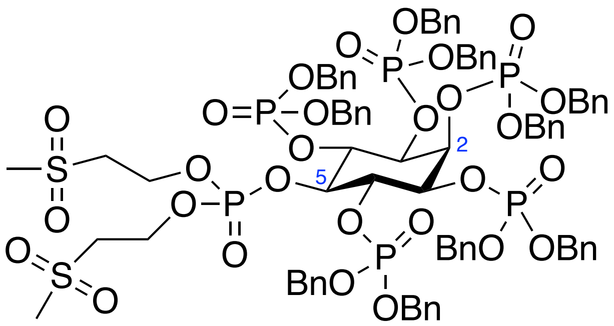

myo-Inositol-5-bis[2-(methanesulfonyl)ethyl]phosphate (**7**, 110 mg, 0.233 mmol) was dissolved in dry MeCN and the solution concentrated *in vacuo* three times, then the residue was suspended in dry DCM and the solution concentrated again thoroughly to dry the starting material. The starting pentaol (**7**) was then suspended in dry DCM (15 mL) and put under argon. To this suspension, 5-phenyl-1*H*-tetrazole (510 mg, 3.49 mmol, 15.0 equiv.) and dibenzyl phosphoramidite (0.8 mL, 2.33 mmol, 10.0 equiv) were added, and the reaction mixture was allowed to stir at RT for 16 h. The solution was then cooled to -78 °C before *m*CPBA (858 mg, 70%, 3.49 mmol, 15.0 equiv) was added. The reaction mixture was allowed to warm to RT and was then diluted with EtOAc (150 mL). The solution was washed with 10% aq. Na_2SO_3 (2 x 30 mL) and 1.0 M HCl (30 mL), dried over $MgSO_4$ and concentrated *in vacuo*. The residue was purified by flash column chromatography (P.E.-EtOAc, 0-100%) to give the pure product as a colourless oil (329.1 mg, 0.225 mmol, 97% yield).**^{1}HNMR** ($CDCl_3$, 400Hz): 7.32-7.23 (m, 46H, Ar), 7.17-7.15 (m, 4H, Ar), 5.67 (br d, *J*= 9.0 Hz, 1H, H-2), 5.23-5.19 (m, 2H, C*H₂*Ar), 5.14-4.90 (m, 18 H, H-4 and H-6 and C*H₂*Ar), 4.82-4.77 (m, 2H, C*H₂*Ar), 4.54 (q, *J*= 9.5 Hz, 1H, H-5), 4.49-4.43 (m, 2H, MSE CH_2), 4.39-4.28 (m, 4H, H-1 and H-3 and MSE CH_2), 3.19-3.06 (m, 4H, MSE CH_2), 2.76 (s, 6H, SC*H₃*); **^{31}PNMR** ($CDCl_3$, 100 Hz): -1.47 (2P), -1.49 (2P), -2.13, -2.84; **^{13}CNMR** ($CDCl_3$, 100 Hz): 135.8-135.5 (m, Ar), 128.9-128.8 (m, Ar), 128.0 (Ar), 77.4 (C-5), 75.6 (C-2), 74.7 (C-4 and C-6), 73.3 (C-1 and C-3), 70.4-70.0 (m, *C*H_2Ar), 62.21 (MSE CH_2), 62.17 (MSE CH_2), 54.5 (MSE CH_2), 54.4 (MSE CH_2), 42.5 (S*C*H_3); **HRMS (ESI)**: *m/z* calcd $[M+H]^+$ for $C_{82}H_{90}O_{28}P_6S_2$: 1773.3558, found: 1773.3518.

5-Diphospho-*myo*-inositol-1,2,3,4,6-pentakisphosphate triethylammonium salt (1)

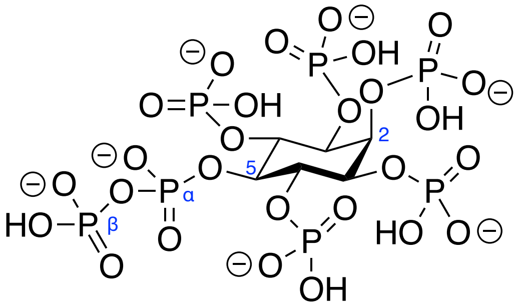

myo-Inositol-1,2,3,4,6-pentakis(dibenzyl)phosphate-5-bis[2-(methanesulfonyl)ethyl]-phosphate (**8**, 65.2 mg, 0.045 mmol) was dissolved in $CDCl_3$ (2 mL) in an NMR tube, and to this solution DBU (25 µL, 177 µmol, 4.0 equiv.) followed by BSTFA (114 µL, 461 µmol, 10.3 equiv.) were added. ^{31}PNMR was used to ensure the silylation was complete with singlets at -0.97 (2P), -1.70 (2P), -2.42 (1P) and a diagnostic singlet at -17.51 (1P). Methanol (26 µL, 2.05 mmol) was then added to the reaction and, after 3 min, TFA (27 µL, 0.347 mmol) was added. ^{31}PNMR spectroscopy confirmed that desilylation was completed with signals at 2.64 (1P), -1.62 (2P) and -1.94 (3P). The reaction mixture was concentrated and the residue was thoroughly dried on a vacuum line. This crude residue was then dissolved in $CDCl_3$, and to this solution 5-phenyl-1*H*-tetrazole (42.9 mg, 0.29 mmol, 6.6 equiv.) and dibenzyl phosphoramidite (41 µL, 0.12 mmol, 2.7 equiv.) were added. After an hour, an additional portion of phosphitylating agent (41 µL, 0.12 mmol, 2.7 equiv.) was added, and the reaction mixture was allowed to stir for another 2h. ^{31}PNMR spectroscopy confirmed successful phosphitylation with a signal at 139.6 (β-P). The reaction mixture was then cooled to -78 °C, before *m*CPBA (13 mg, 70%, 0.052 mmol, 2 equiv) was added. The solution was allowed to warm to RT, before being concentrated *in vacuo* without heat. This residue (**9**) was purified by column chromatography (EtOAc and MeOH, 0-20%), before being immediately deprotected *via* hydrogenation (the compound was not studied at this stage as the product proved to be unstable following purification).

The protected pyrophosphate (assumed **9**, 40.8 mg, 0.0224 mmol) was dissolved in a mixture of THF (4.6 mL), MeOH (3 mL) and 1M TEAB (2.1 mL). To this solution, $Pd(OH)_2/C$ (20%, ≥50% wet, 27.2 mg) was added. The flask was evacuated and flushed with hydrogen three times before leaving the solution to stir in an atmosphere of hydrogen. It was allowed to stir at RT under hydrogen for 3 days. The crude product was purified on an RP-18 column, and the product was identified in the column fractions using a Briggs qualitative phosphate assay. The product fractions were concentrated *in vacuo* without heat, lyophilised and product collected as an off-white glass (21.7 mg, calculated 6.5 TEA counterions per molecule of product, 0.0155 mmol, 69% yield estimated for the hydrogenation step, 35% yield for the complete pyrophosphorylation sequence). **^{1}HNMR** (d4-MeOH, 400 Hz): 4.97 (dt, *J*= 2.9, 10.4Hz, 1H, H-2), 4.60 (q, *J*= 9.7 Hz, 2H, H-4 and H-6), 4.35-4.25 (m, 3H, H-1, H-3 and H-5), 3.15 (s, approx. 39H, TEA CH_2), 1.30 (s, approx. 59H, TEA CH_3); **^{31}PNMR** (d4-MeOH, 162 Hz): 0.87 (2P), 0.68 (2P), -0.29, -10.70 (d, *J*= 22.8 Hz, pyrophosphate β-P), -11.81 (d, *J*= 22.8 Hz, pyrophosphate α-P); **^{13}CNMR** (d4-MeOH, 100 Hz): 79.4 (C-5), 77.5 (C-4 and C-6), 76.8 (C-2), 75.5 (C-1 and C-3), 47.2 (TEA CH_2), 9.3 (TEA CH_3); **HRMS (ESI)**: *m/z* calcd $[M-H]^-$ for $C_6H_{19}O_{27}P_7$: 738.8204, found: 738.8206.

NMR Spectra of Compounds

2-*O*-Benzyl-1,6:3,4-bis-*O*-(2,3-dimethoxybutane-2,3-diyl)-*myo*-inositol (3)

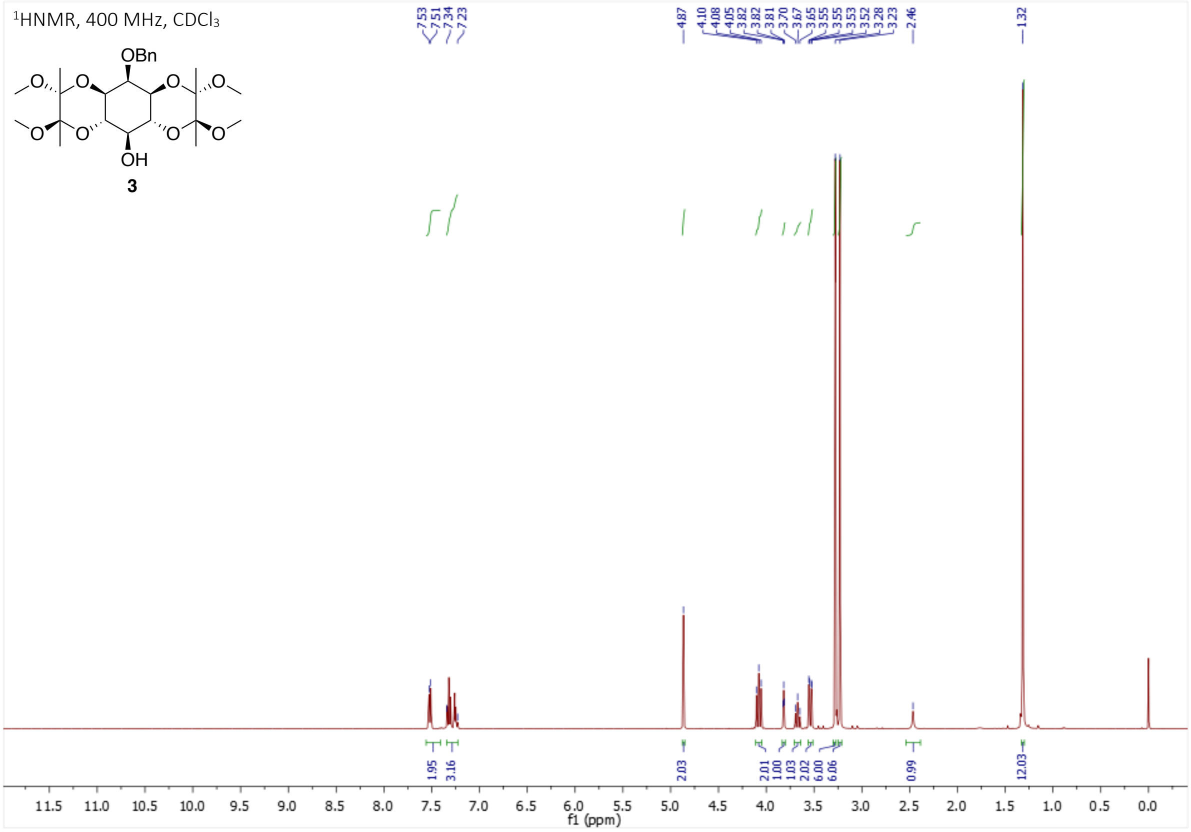

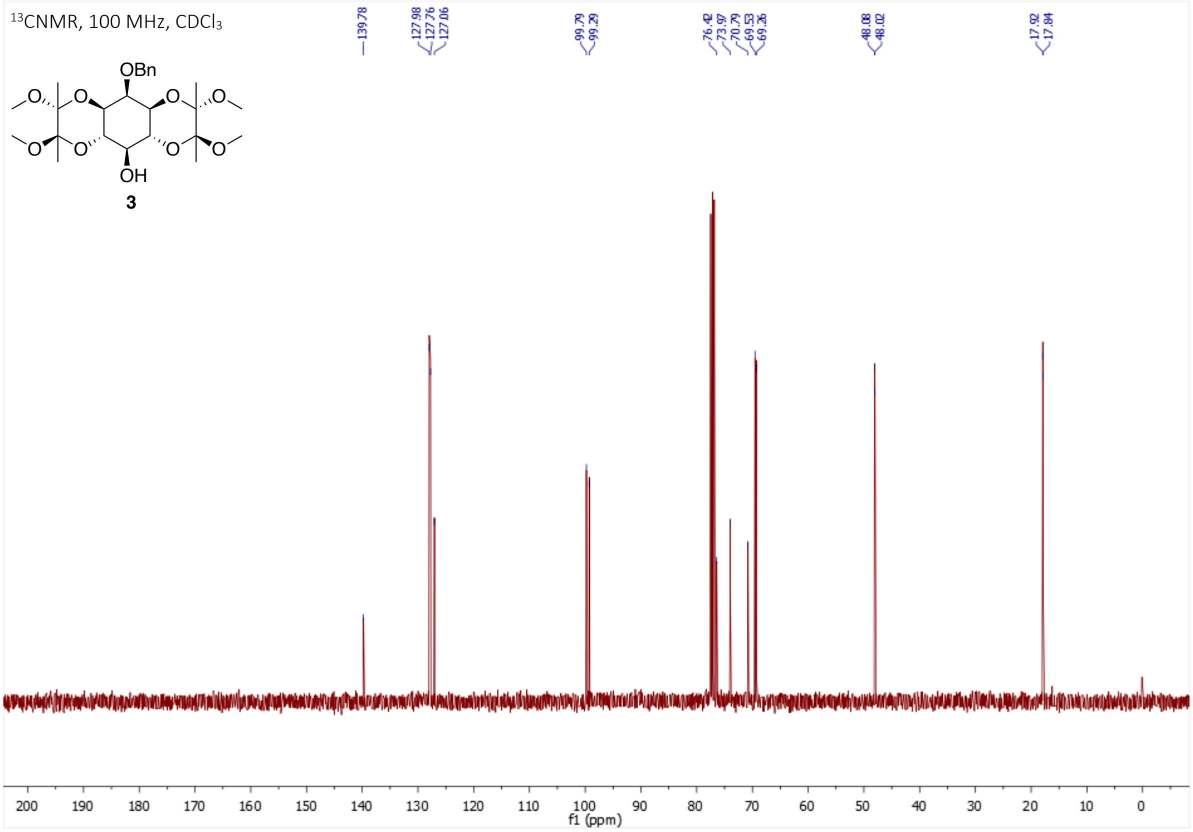

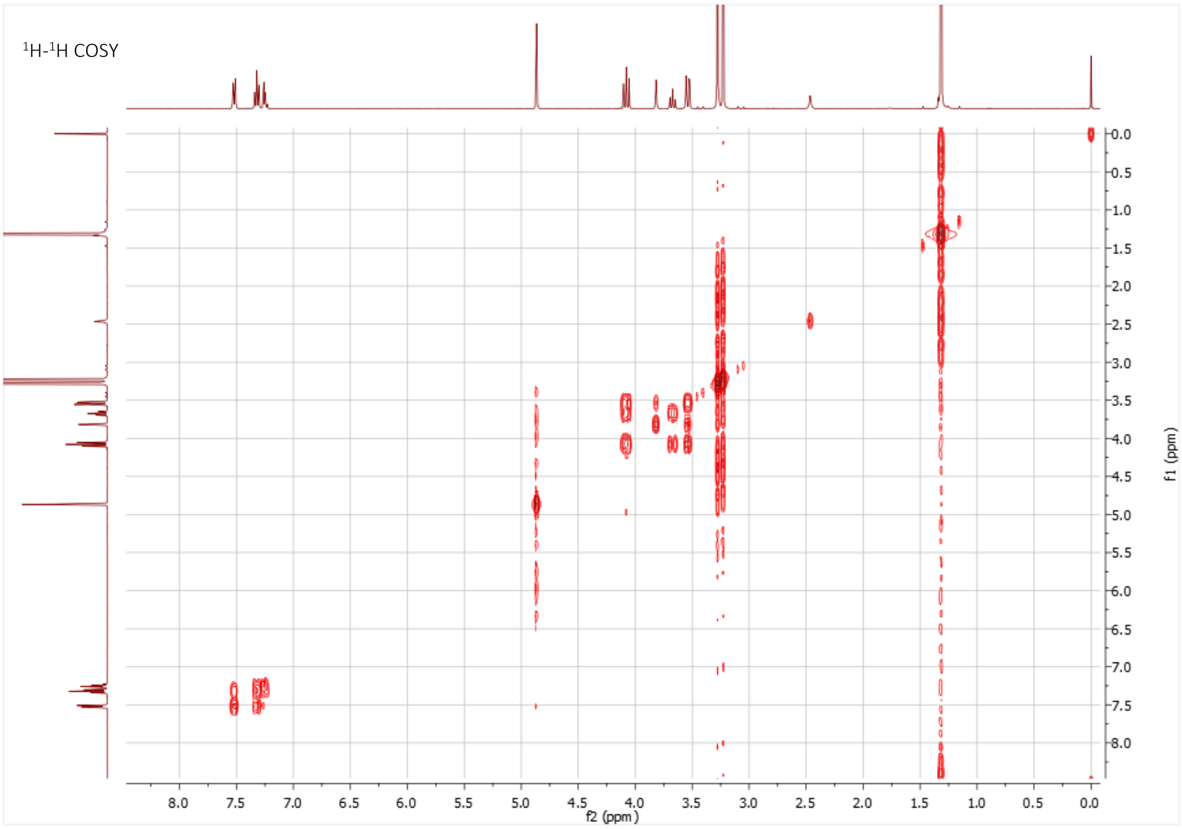
^{1}H-^{1}H COSY
f2 (ppm)
f1 (ppm)

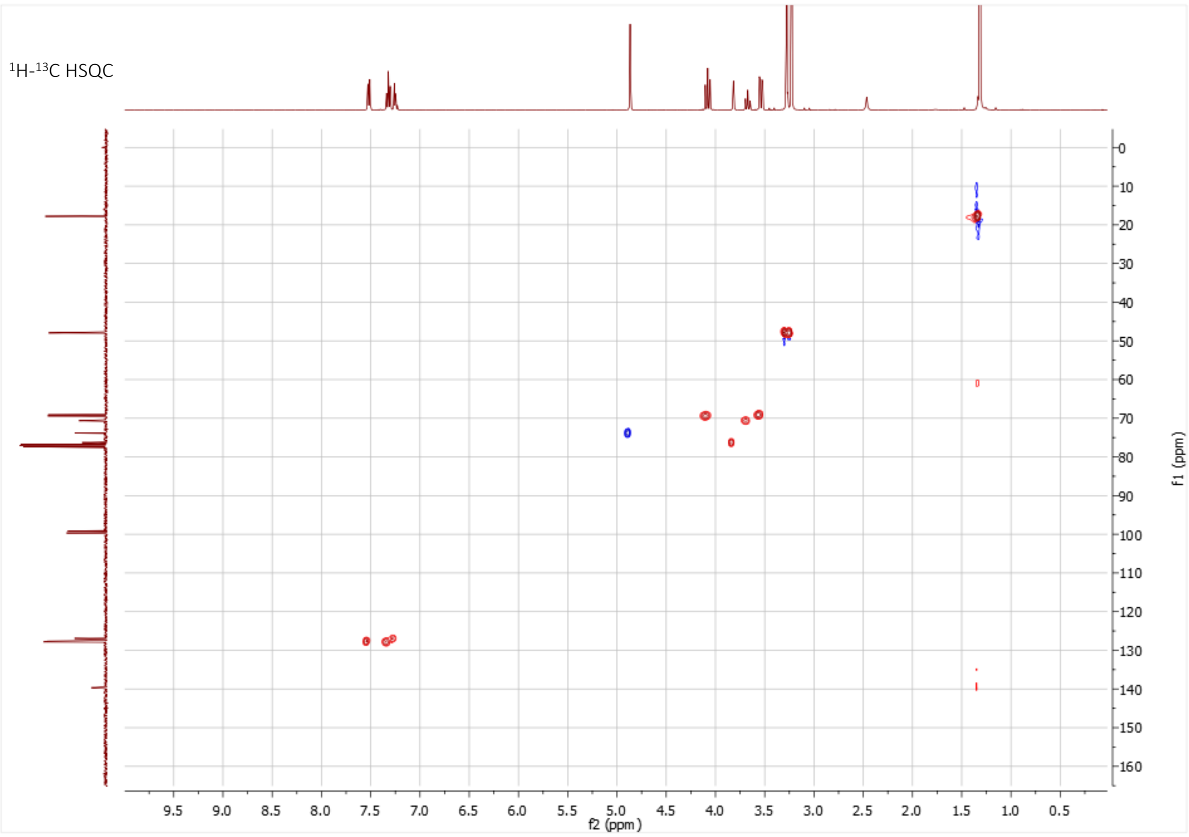
^{1}H-^{13}C HSQC
f2 (ppm)
f1 (ppm)

***N,N*-Diisopropylamino-bis-[2-(methylsulfonyl)ethyloxy]phosphine (4)**

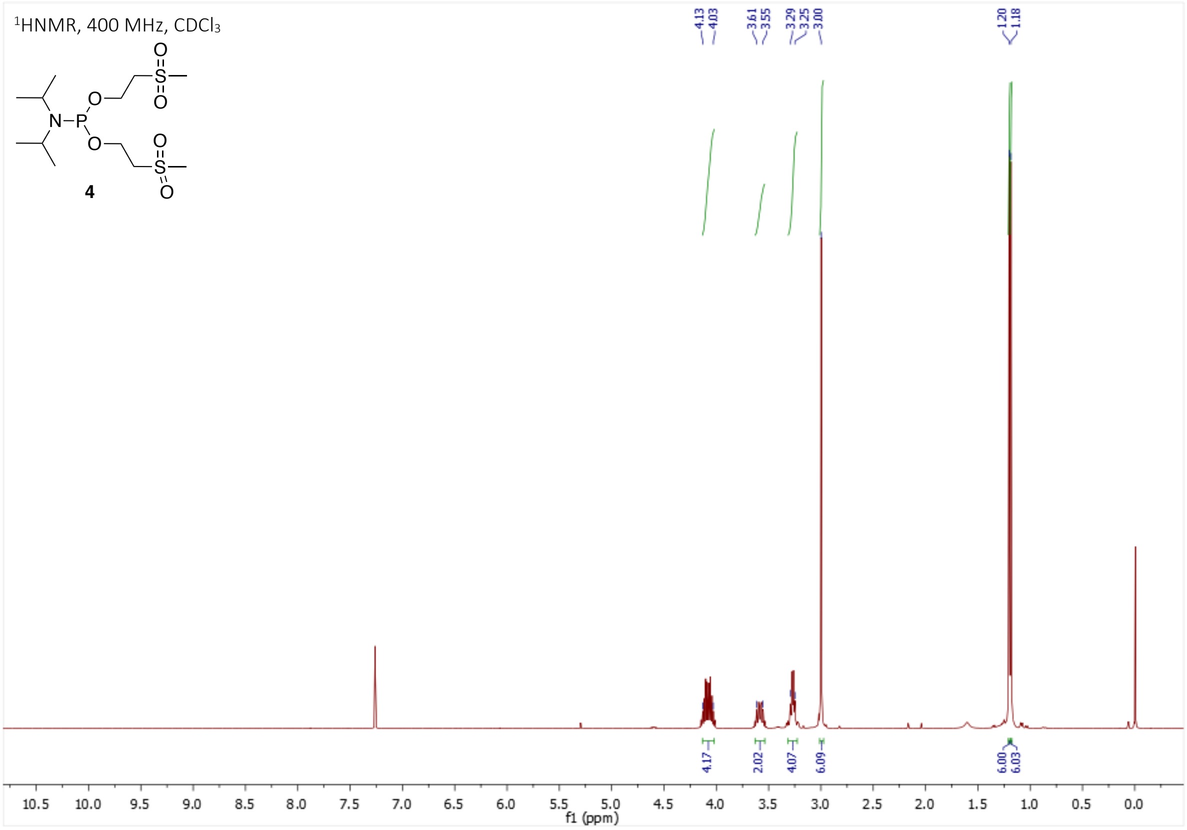

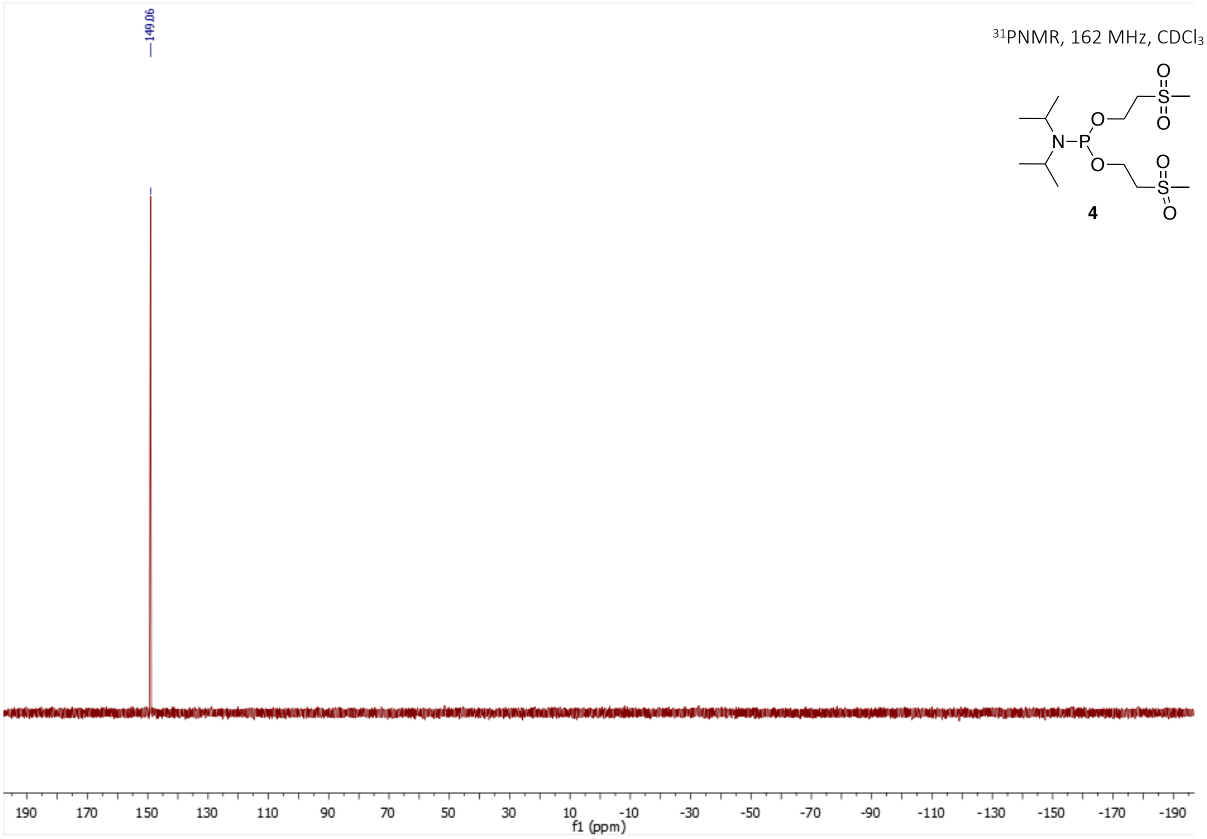

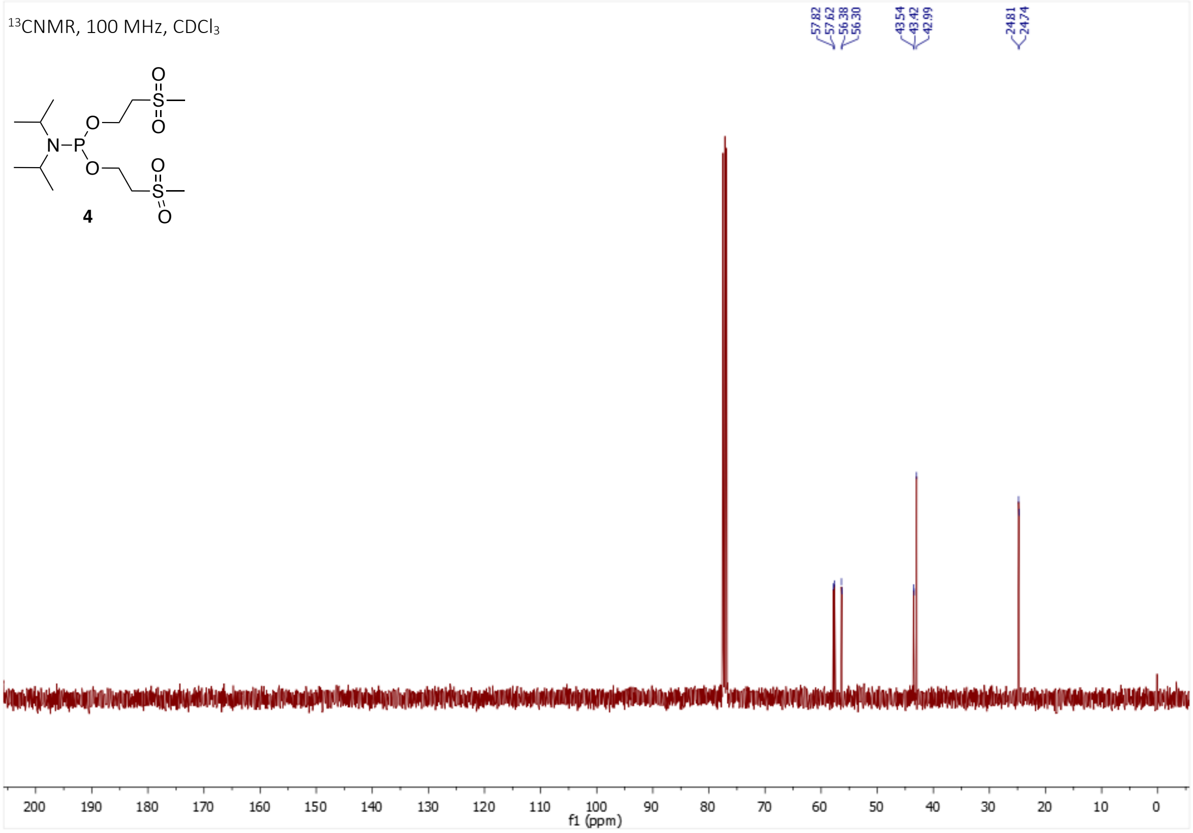

^{13}CNMR, 100 MHz, $CDCl_3$
57.82
57.62
56.38
56.30
43.54
43.42
42.99
24.81
24.74
4
f1 (ppm)

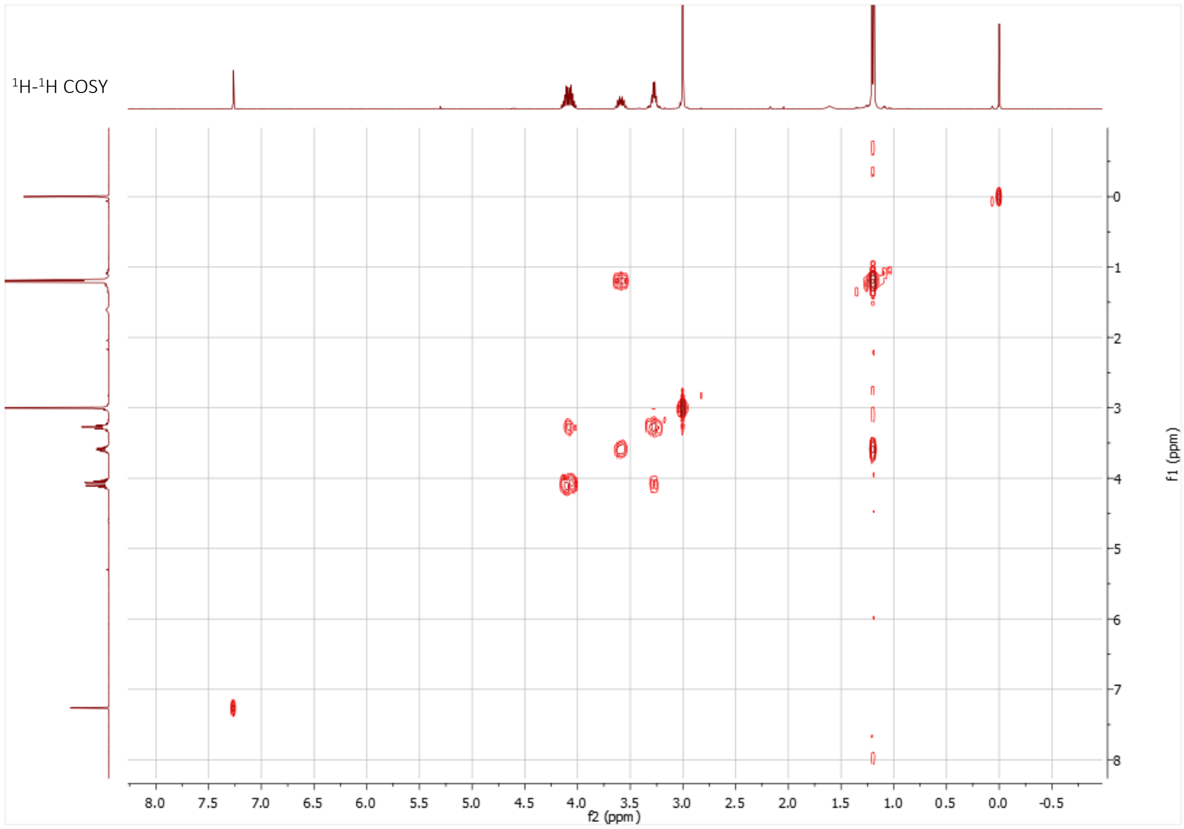
^{1}H-^{1}H COSY
f2 (ppm)
f1 (ppm)

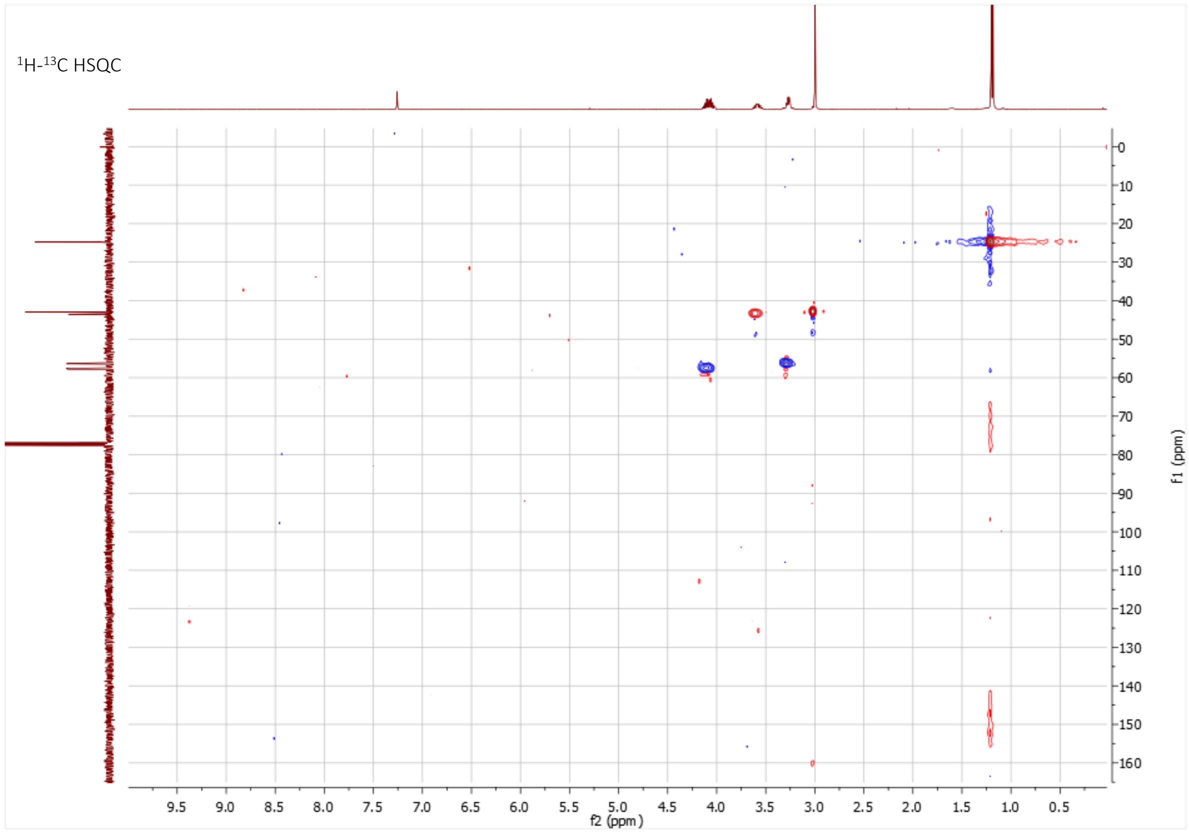
^{1}H-^{13}C HSQC
f2 (ppm)
f1 (ppm)

2-*O*-Benzyl-1,6:3,4-bis-*O*-(2,3-dimethoxybutane-2,3-diyl)-*myo*-inositol-5-bis[2-(methanesulfonyl)ethyl]phosphate (5)

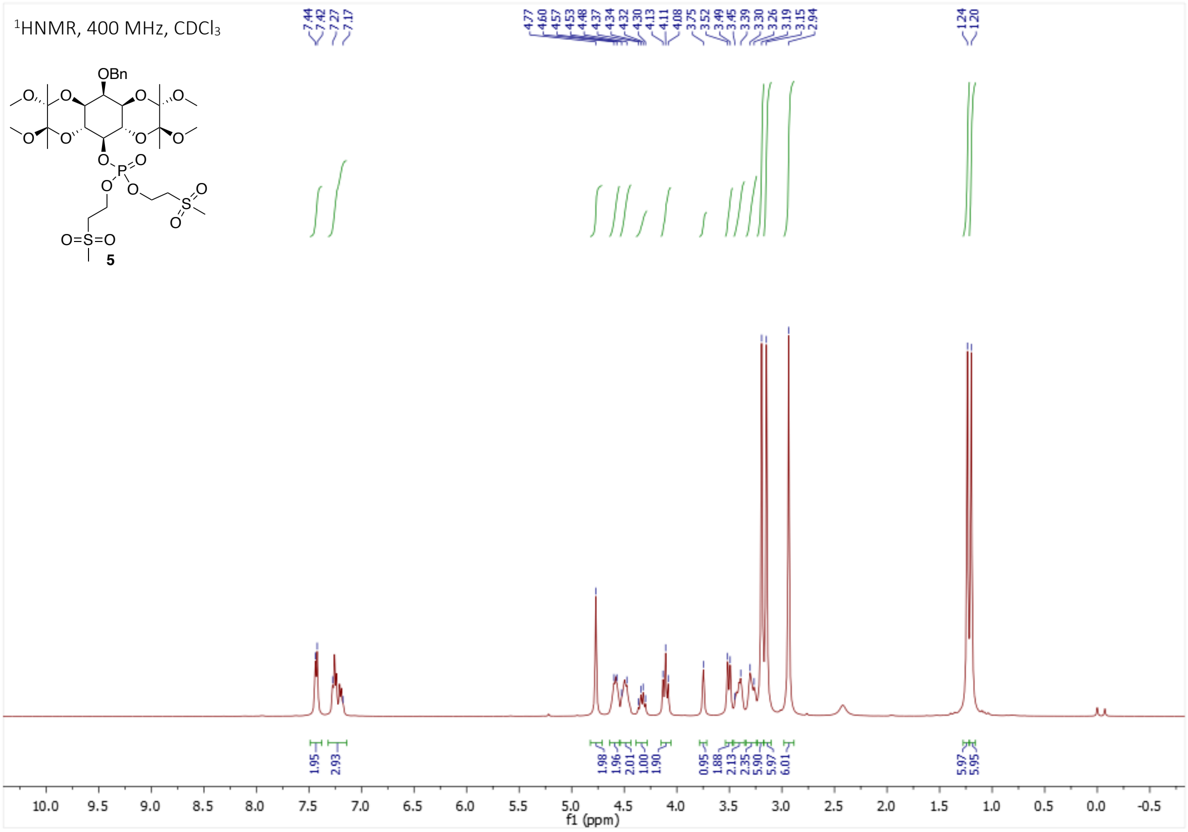

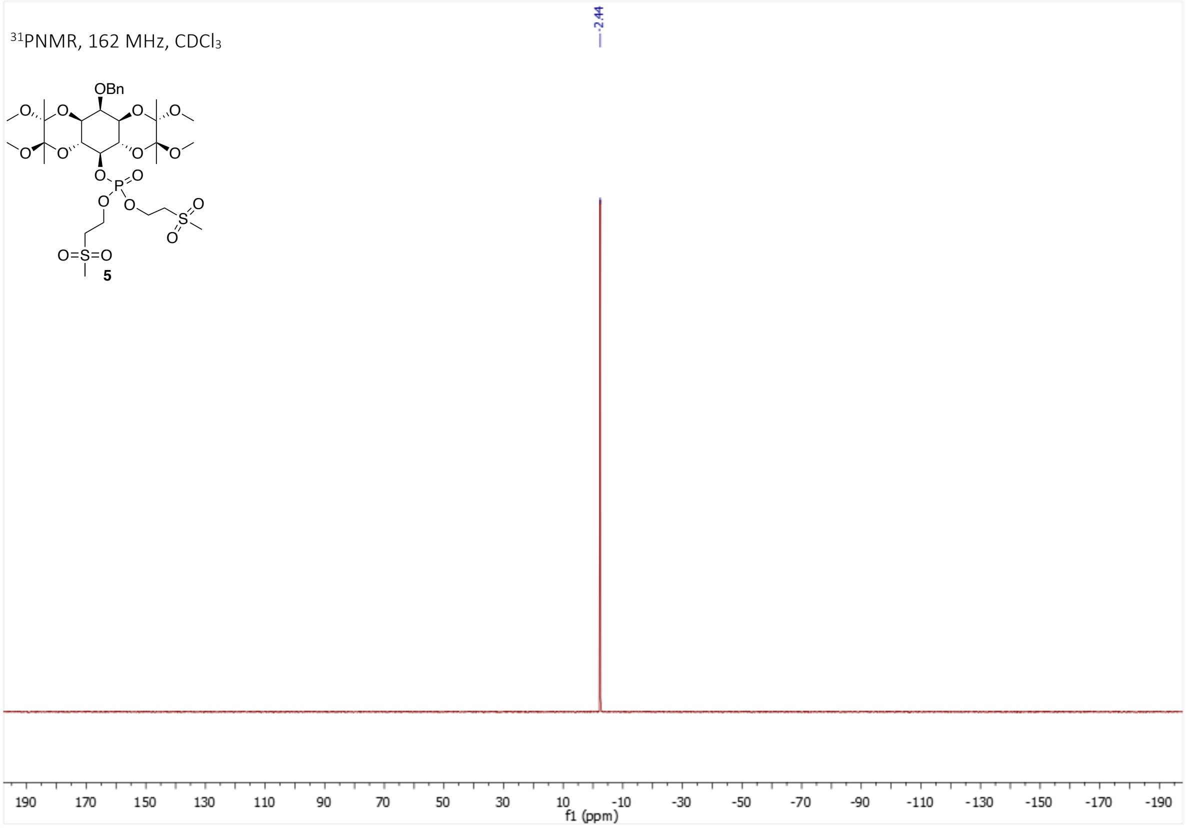

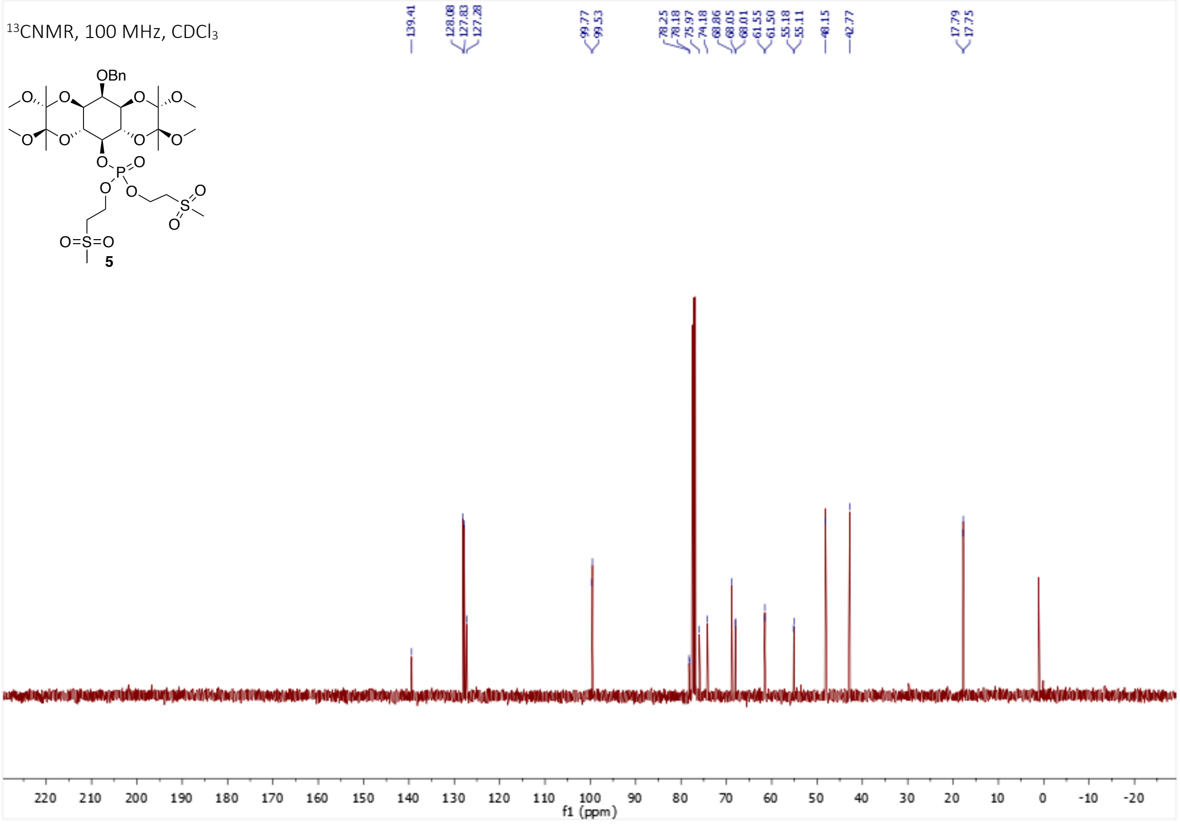
^{13}CNMR, 100 MHz, $CDCl_3$
139.41
128.08
127.83
127.28
99.77
99.53
78.25
78.18
75.97
74.18
68.86
68.05
68.01
61.55
61.50
55.18
55.11
48.15
42.77
17.79
17.75
OBn
5
f1 (ppm)

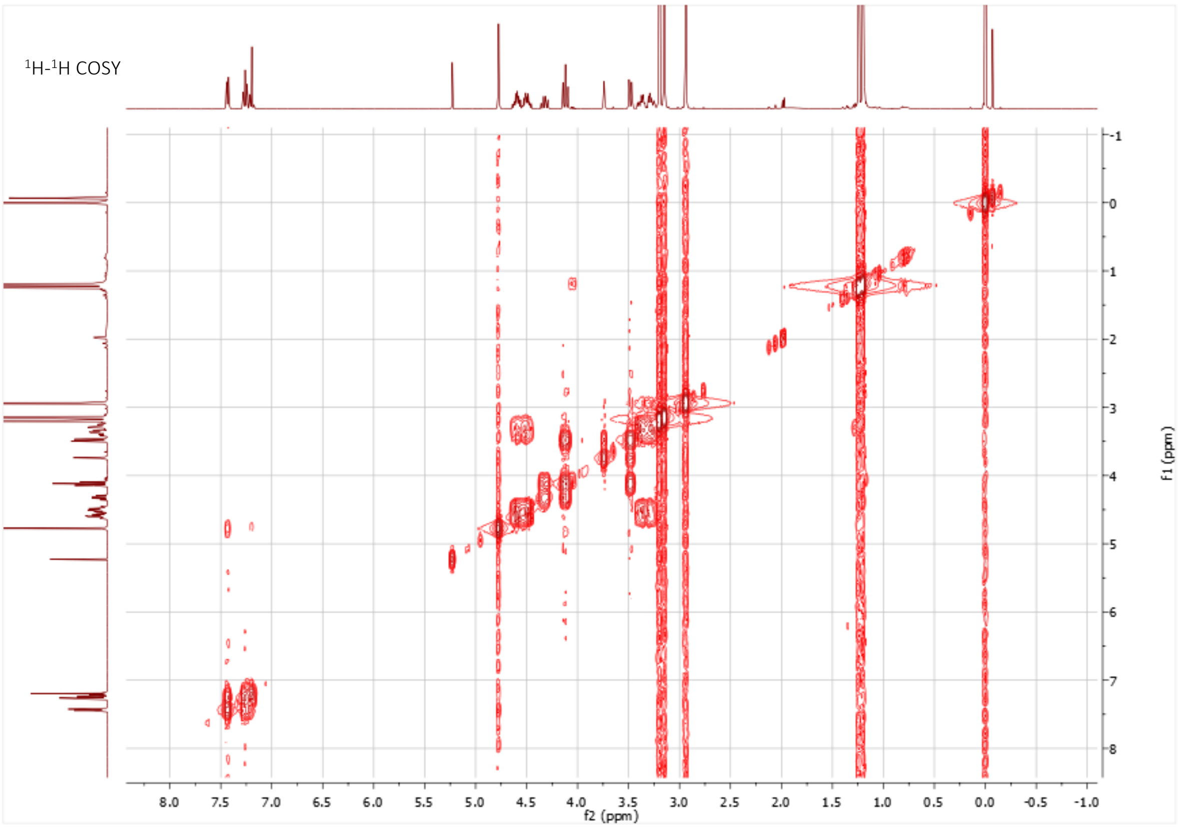
^{1}H-^{1}H COSY
f2 (ppm)
f1 (ppm)

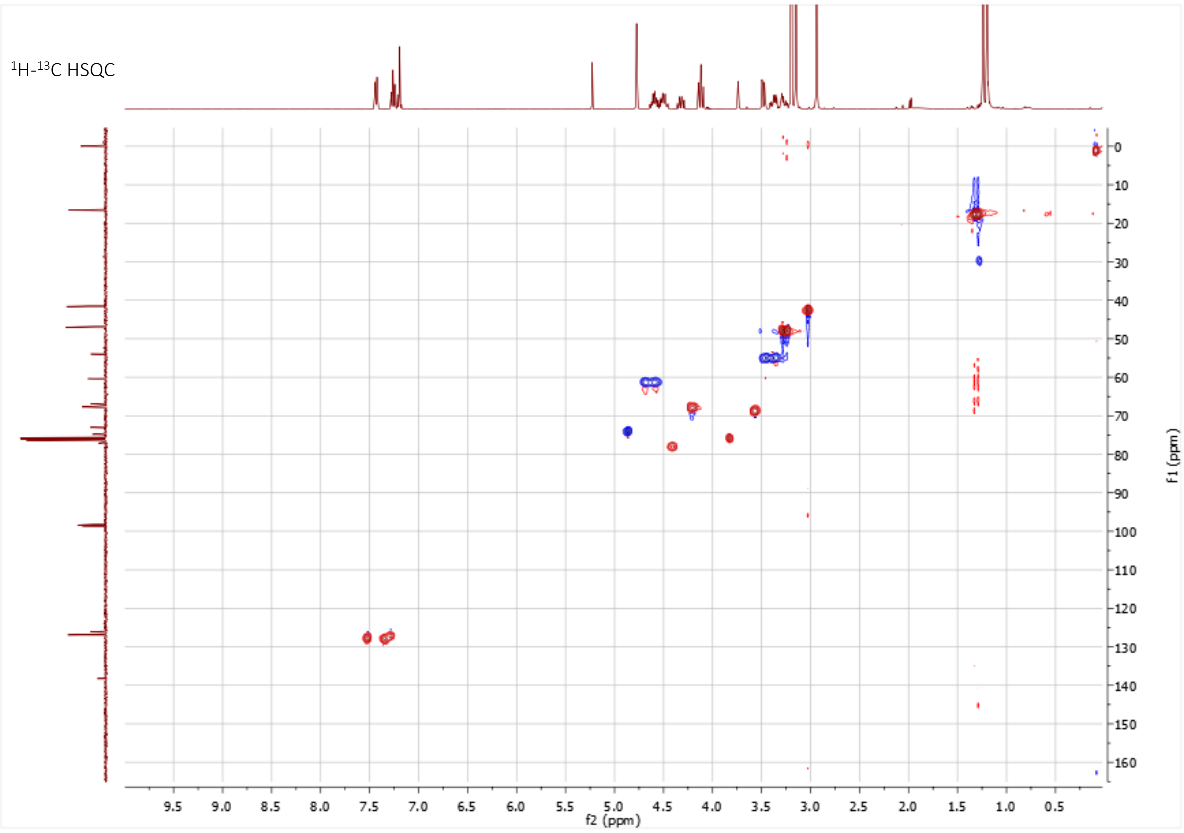

2-*O*-Benzyl-*myo*-inositol-5-bis[2-(methanesulfonyl)ethyl]phosphate (6)

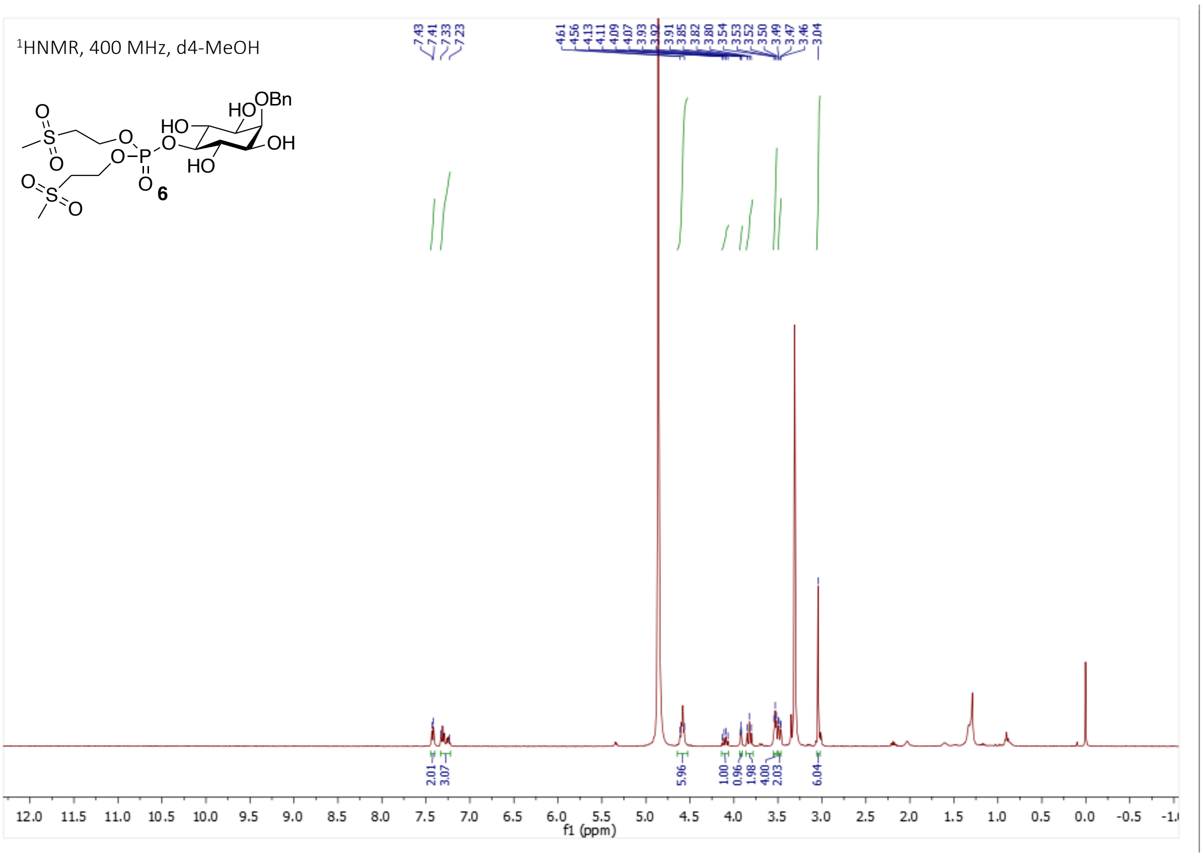

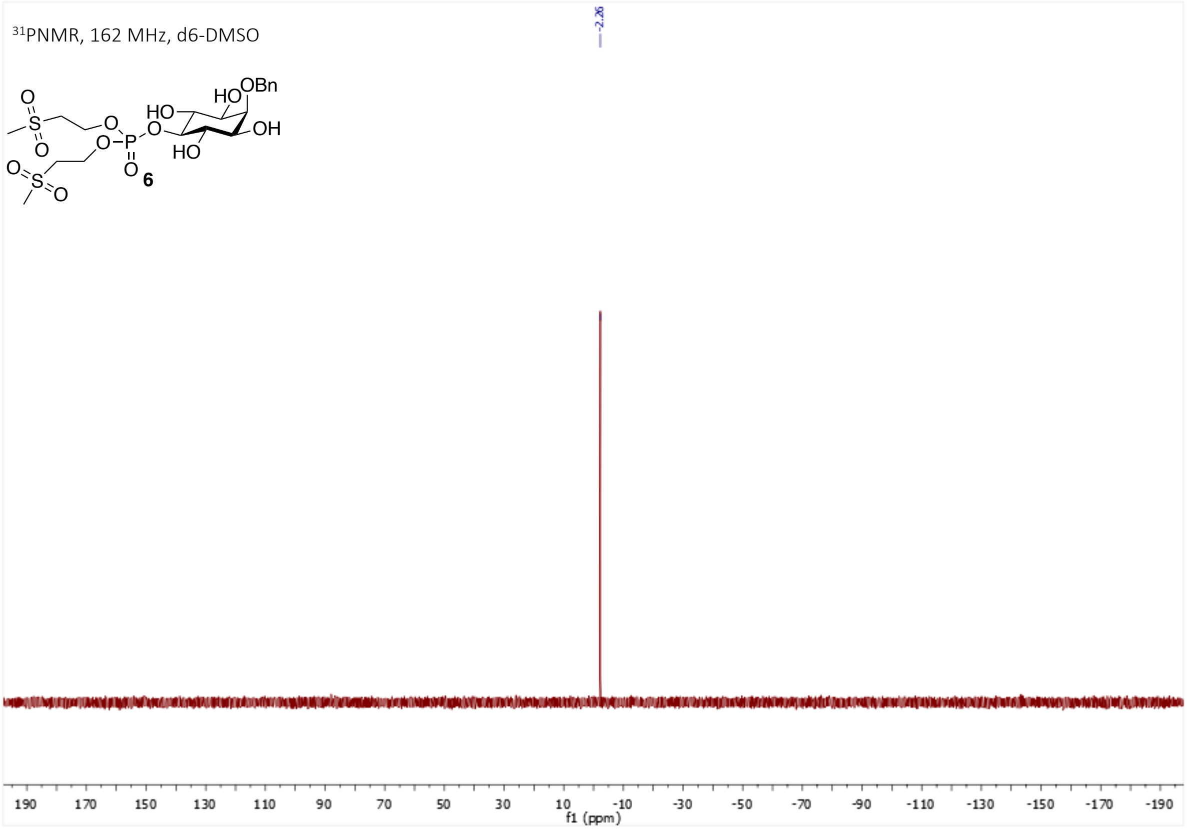
31PNMR, 162 MHz, d6-DMSO
-2.26
6
f1 (ppm)

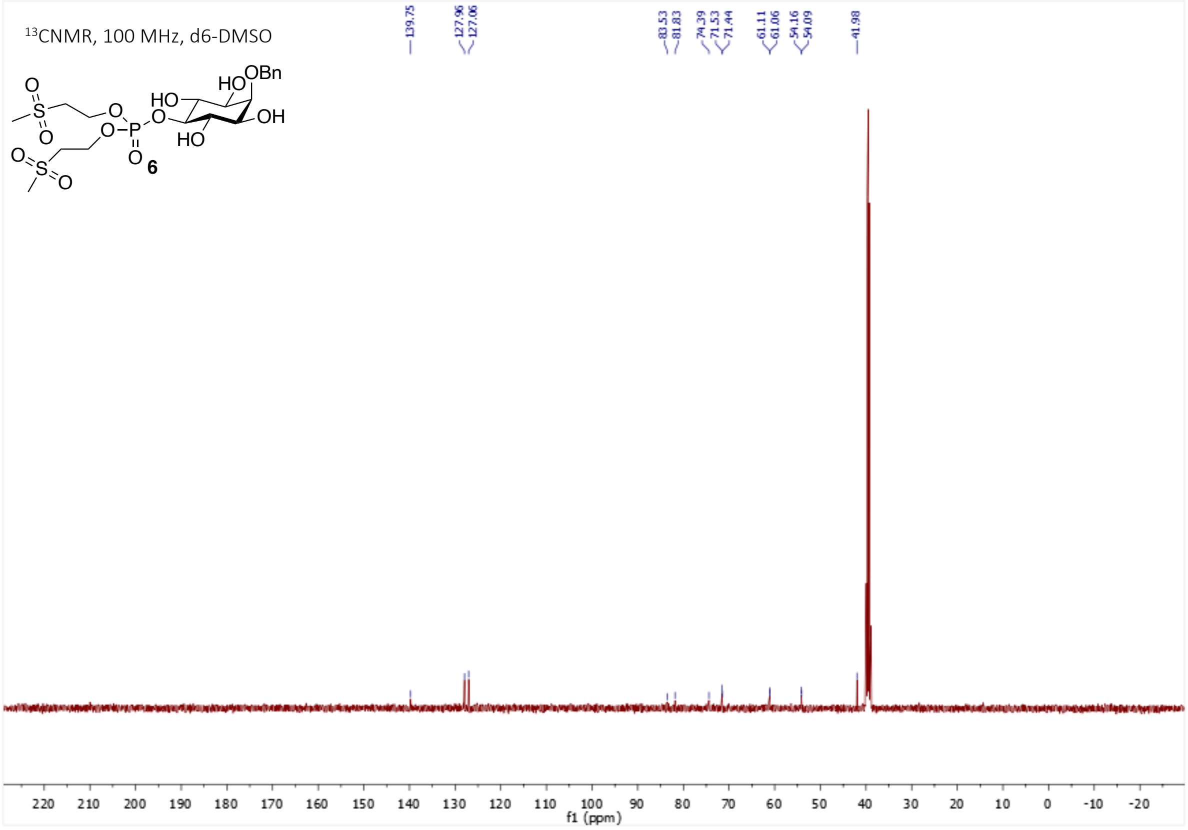
13CNMR, 100 MHz, d6-DMSO
139.75
127.96
127.06
83.53
81.83
74.39
71.53
71.44
61.11
61.06
54.16
54.09
41.98
6
f1 (ppm)

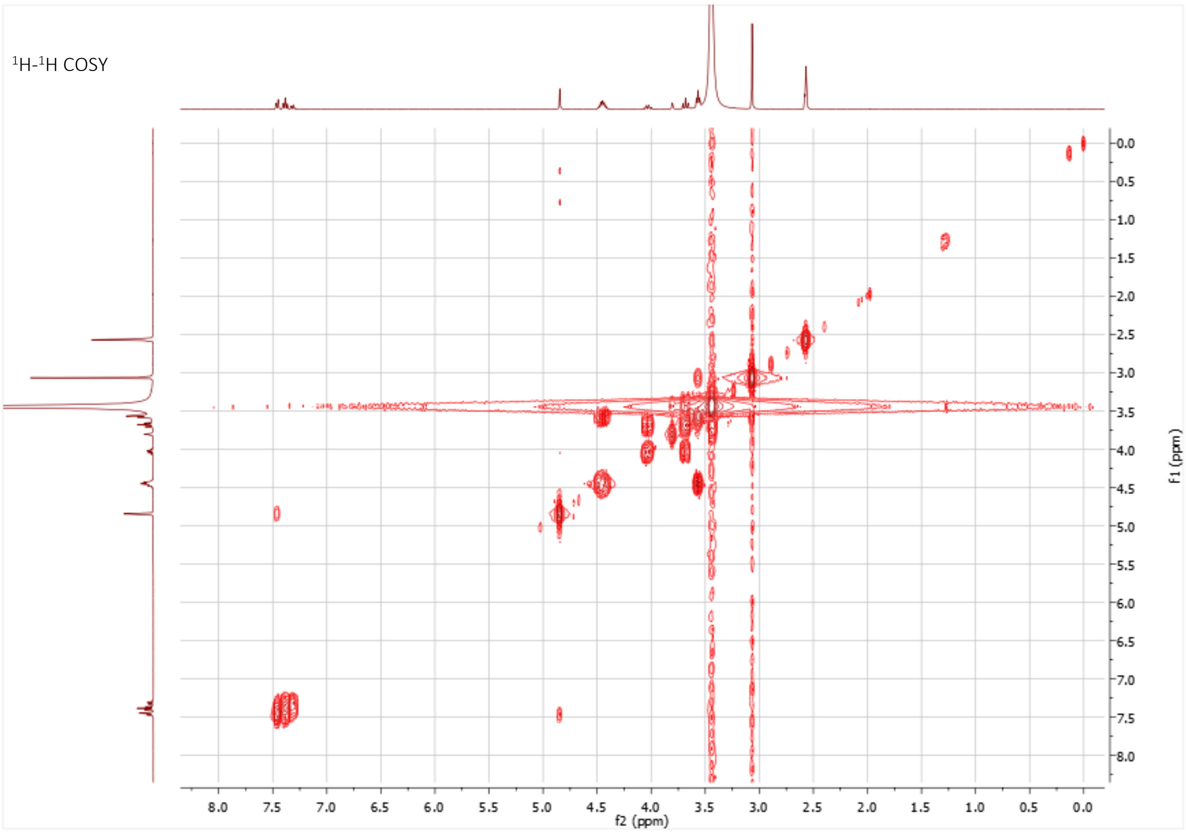

^1H-^1H COSY
f2 (ppm)
f1 (ppm)

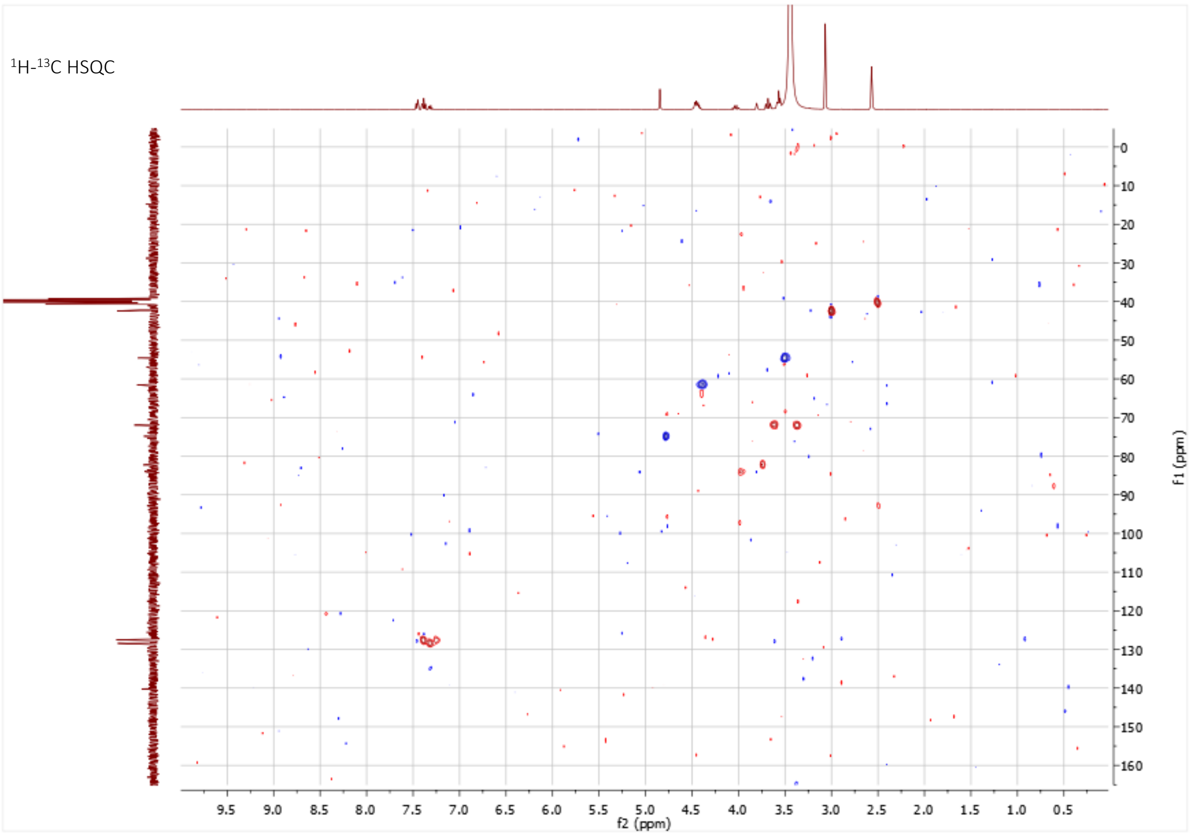

^1H-^{13}C HSQC
f2 (ppm)
f1 (ppm)

***myo*-Inositol-5-bis[2-(methanesulfonyl)ethyl]phosphate (7)**

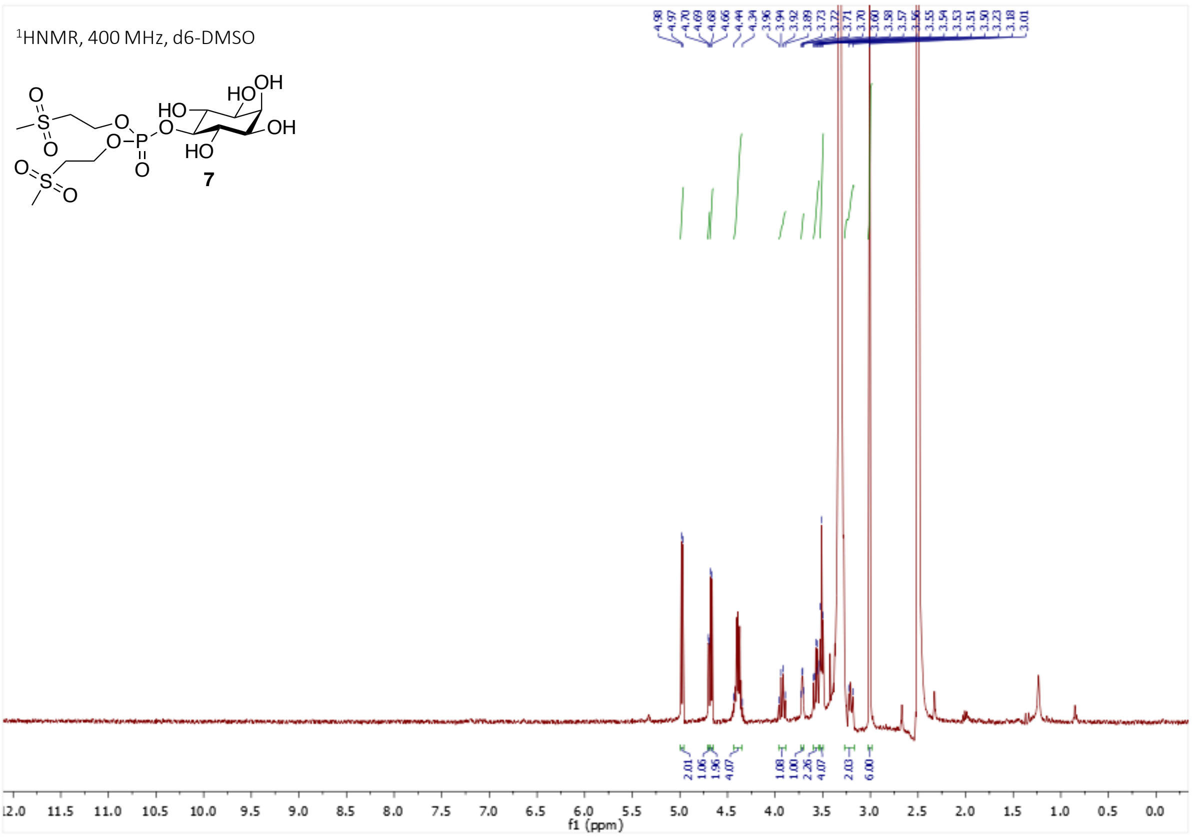

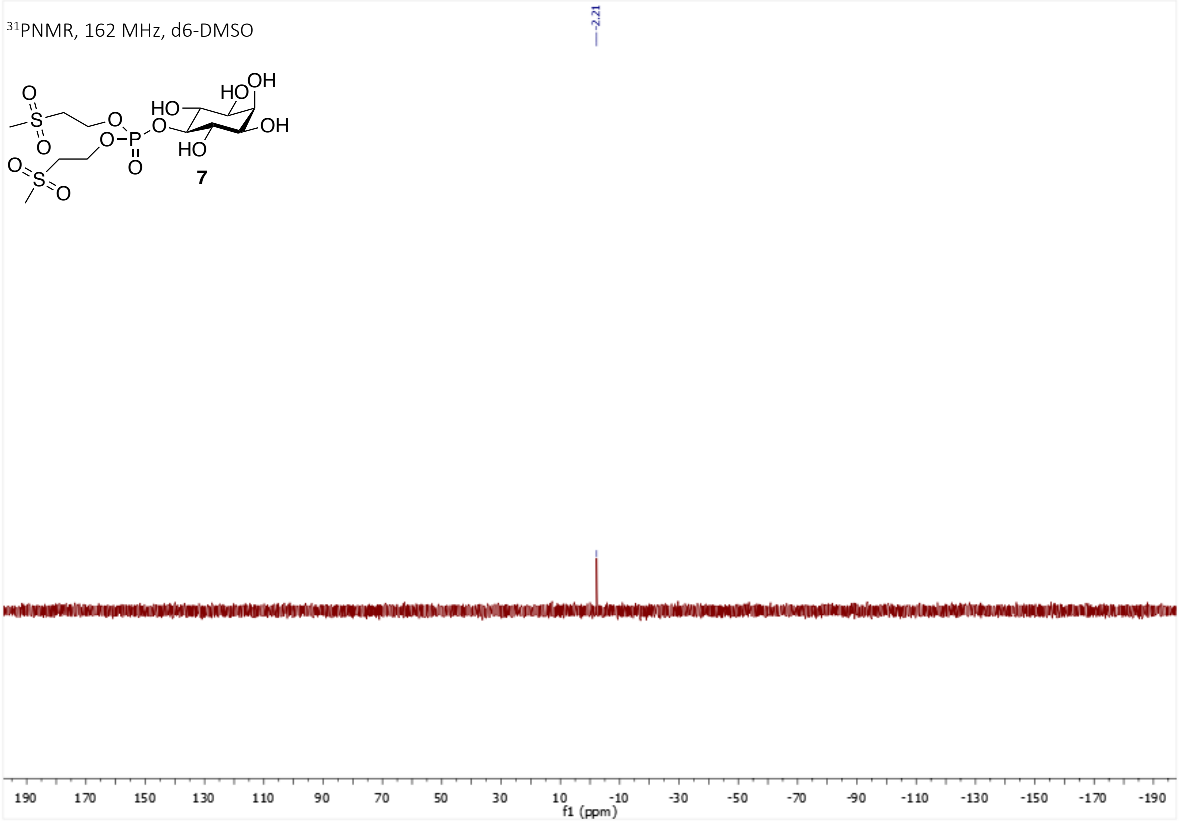

myo-Inositol-1,2,3,4,6-pentakis(dibenzyl)phosphate-5-bis[2-(methanesulfonyl)ethyl]-phosphate (8)

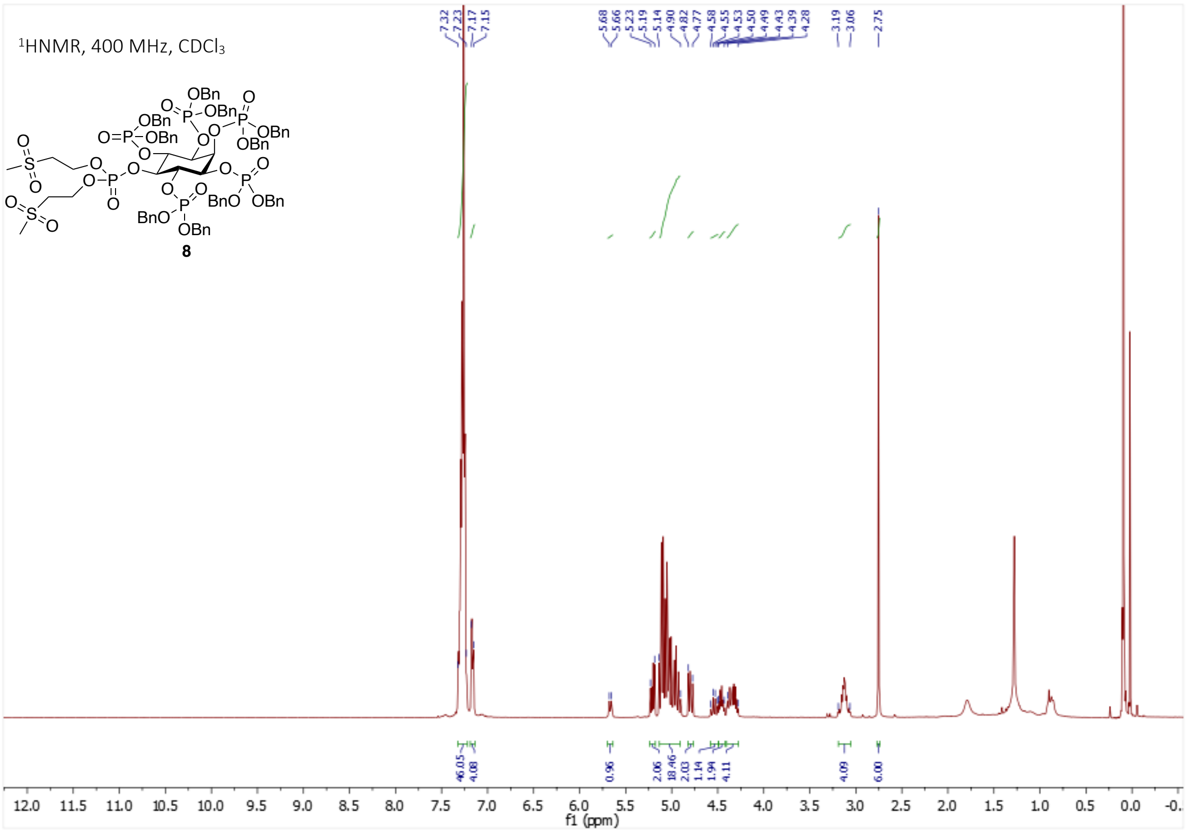

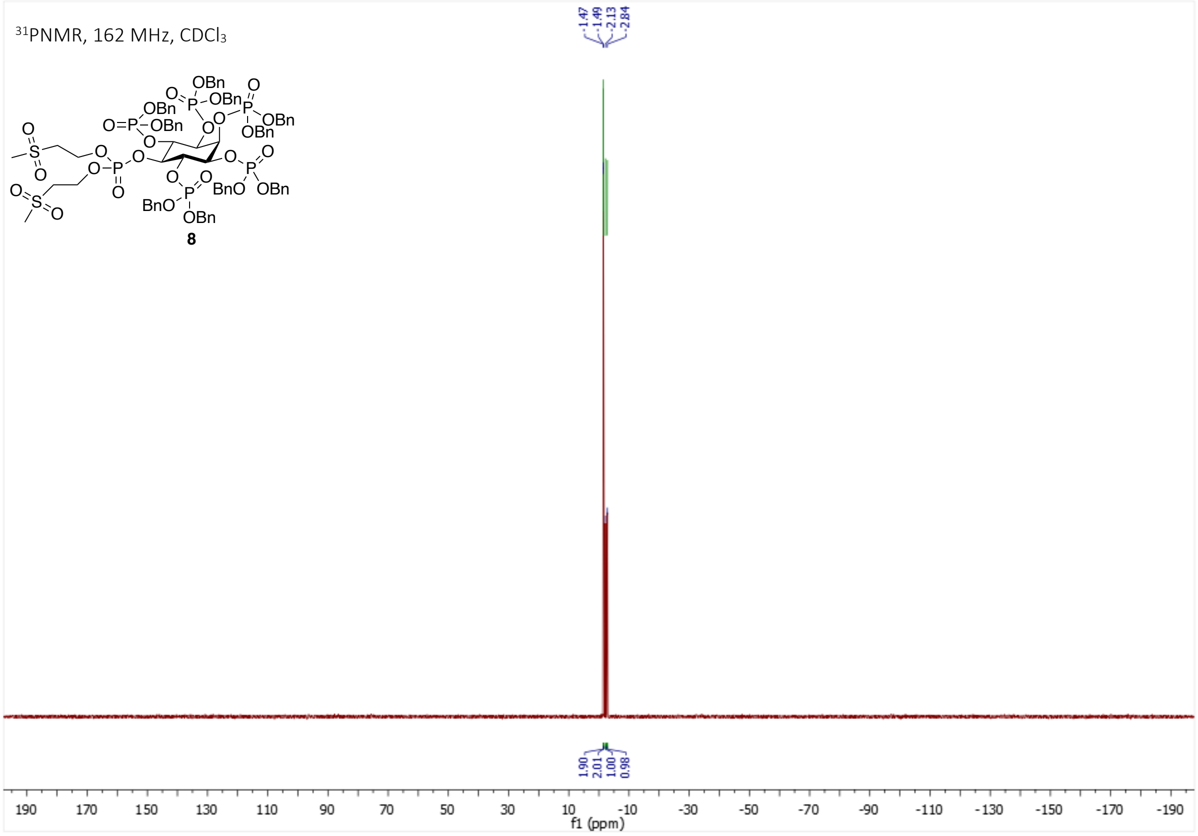

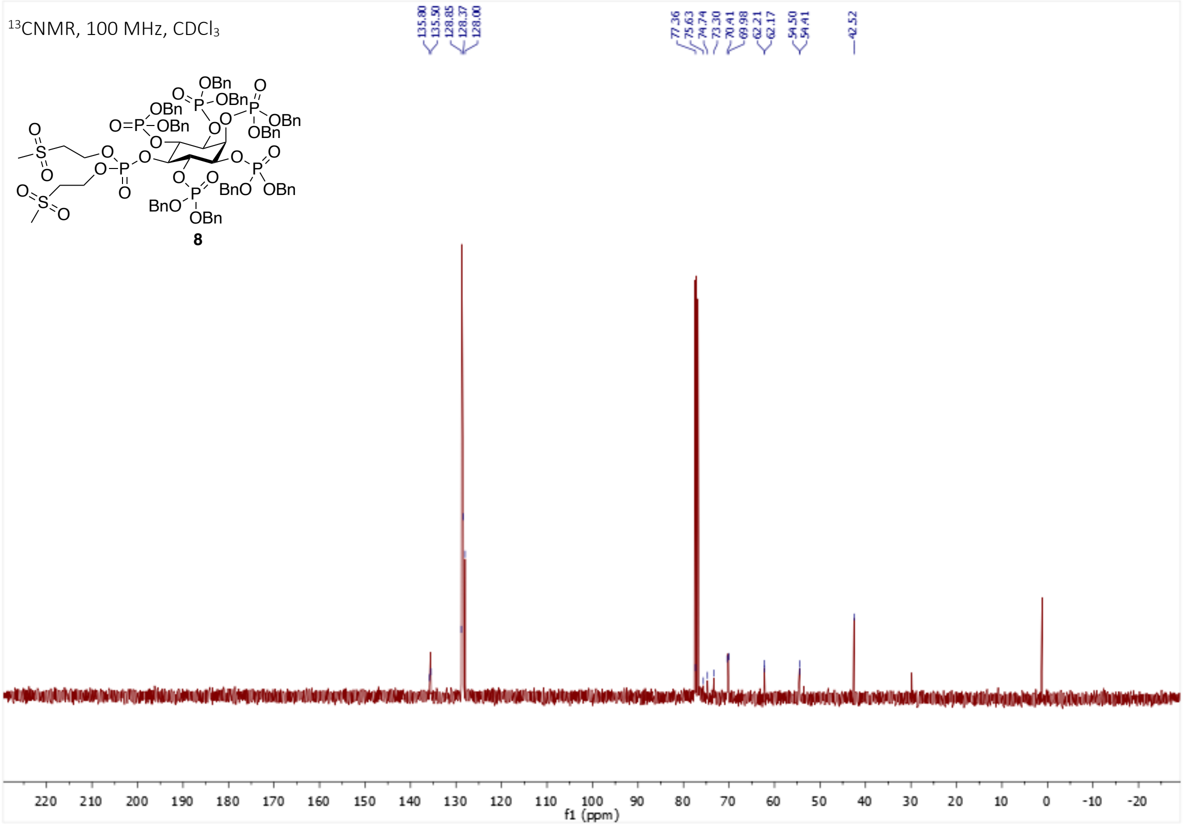

¹³CNMR, 100 MHz, CDCl₃
135.80
135.50
128.85
128.37
128.00
77.36
75.63
74.74
73.30
70.41
69.98
62.21
62.17
54.50
54.41
42.52
8
f1 (ppm)

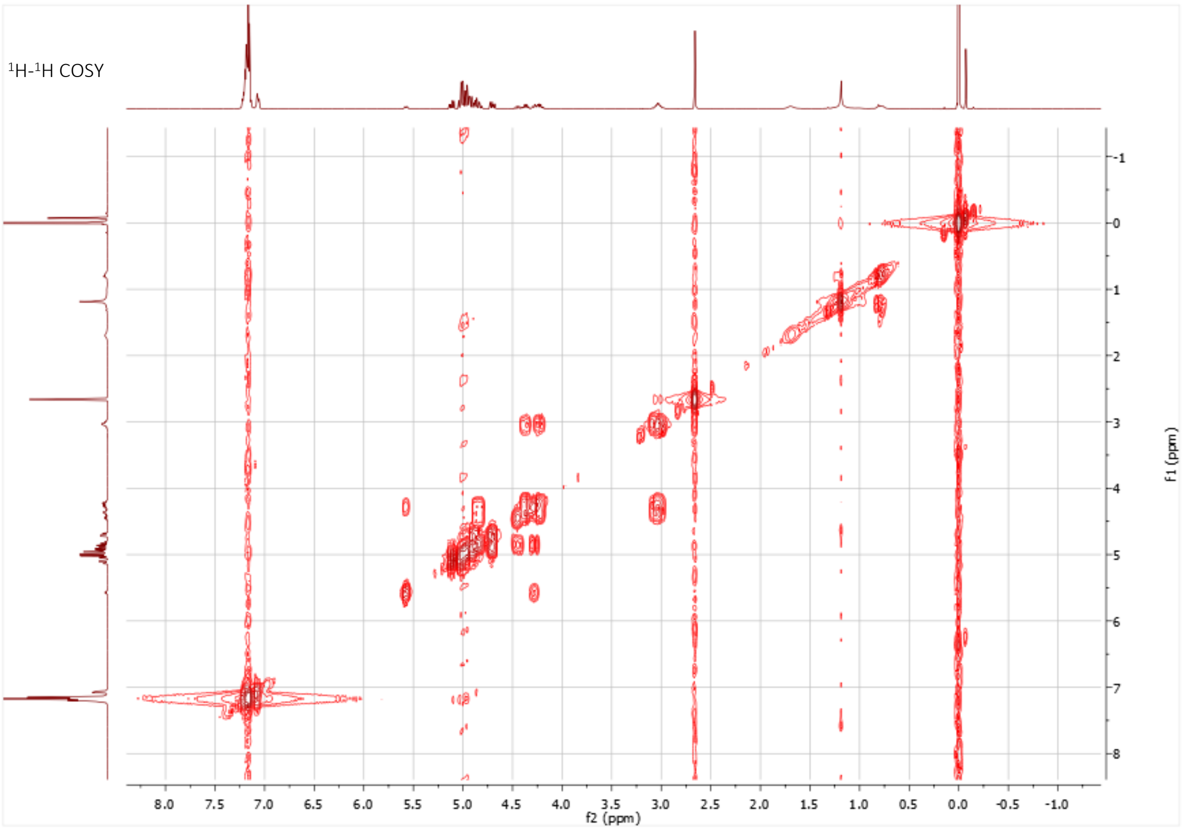

¹H-¹H COSY
f2 (ppm)
f1 (ppm)

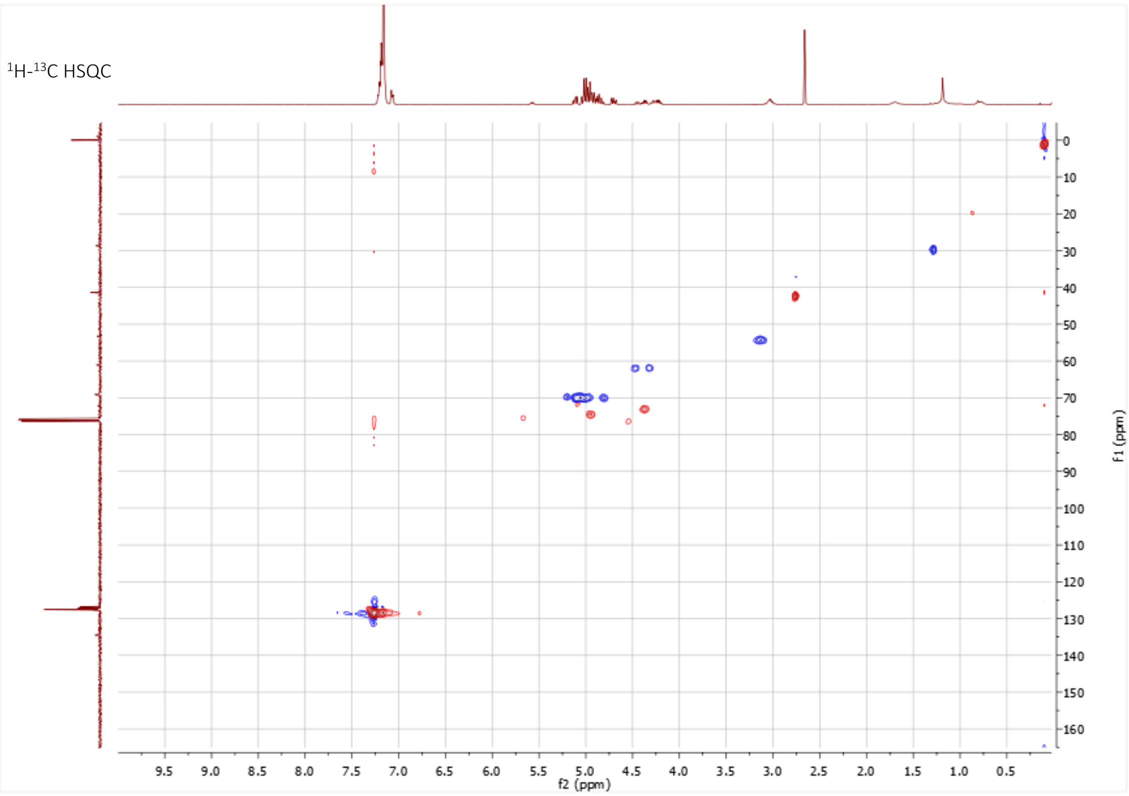

5-Diphospho-*myo*-inositol-1,2,3,4,6-pentakisphosphate triethylammonium salt (1)

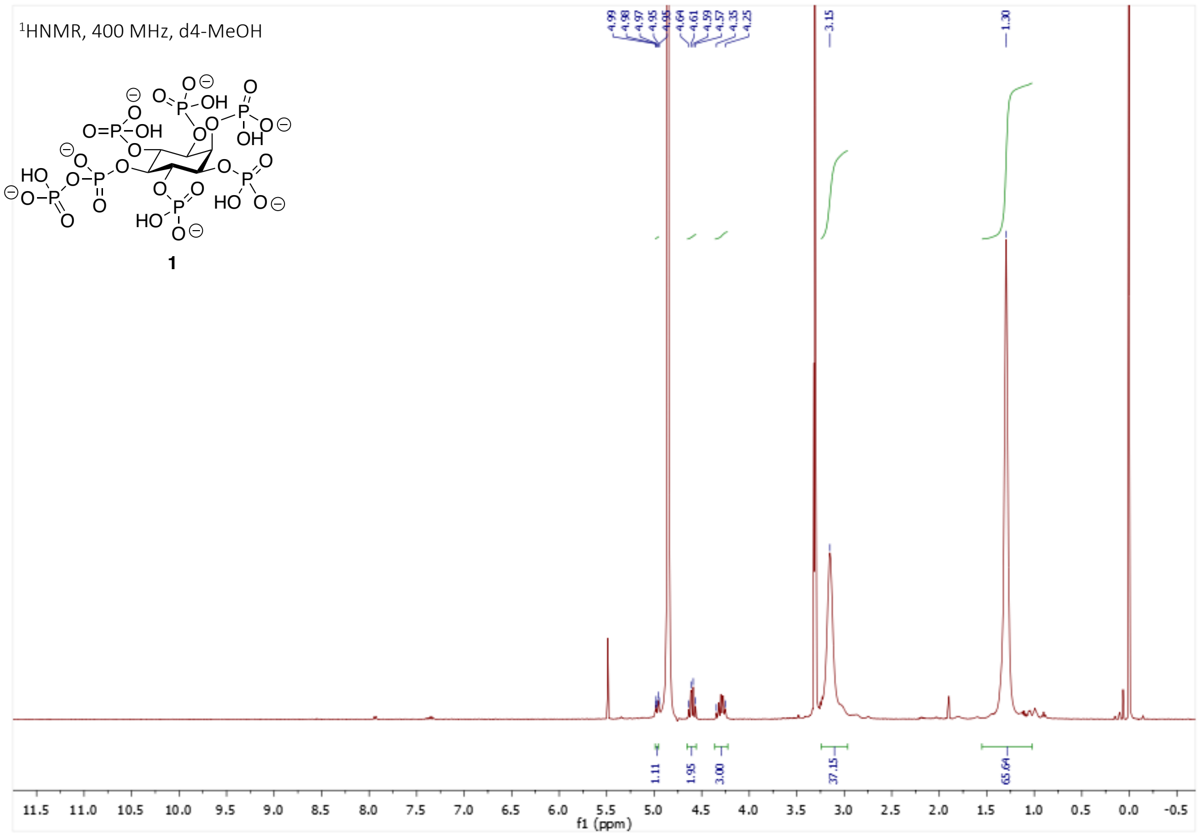

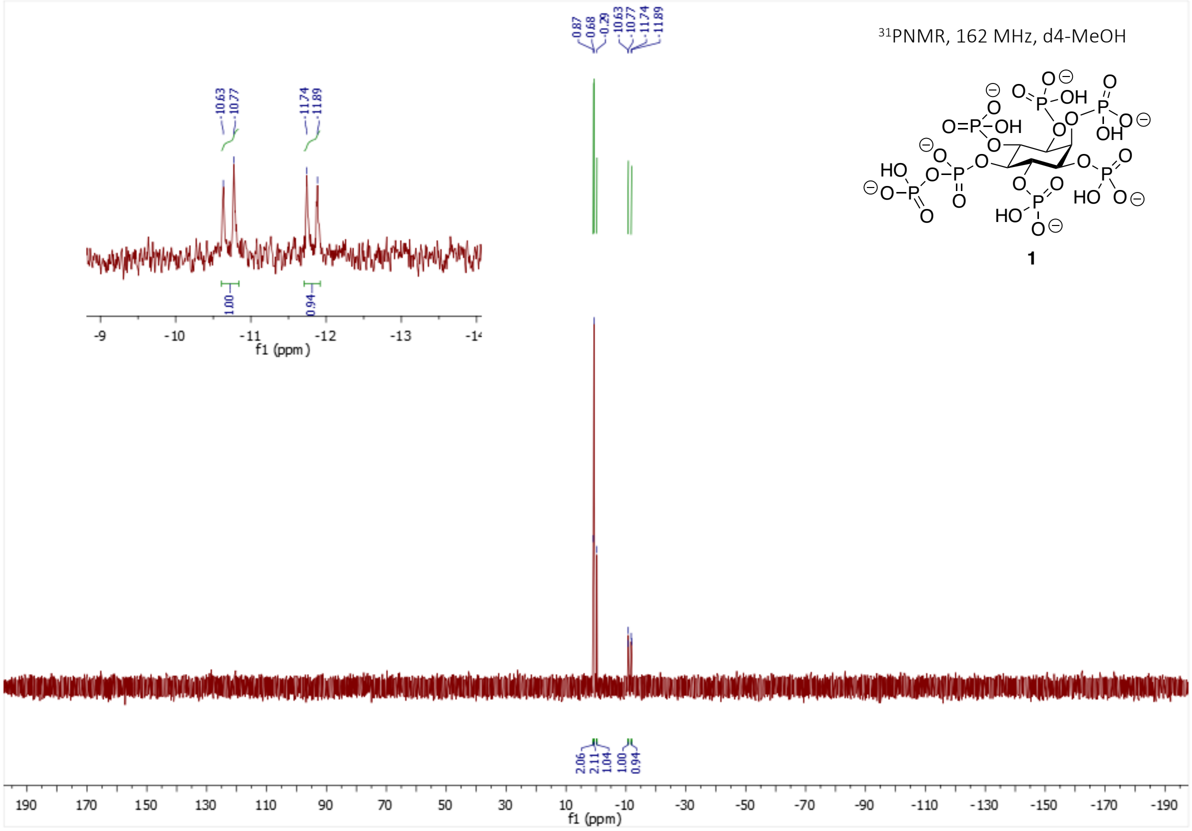
31PNMR, 162 MHz, d4-MeOH
1
f1 (ppm)

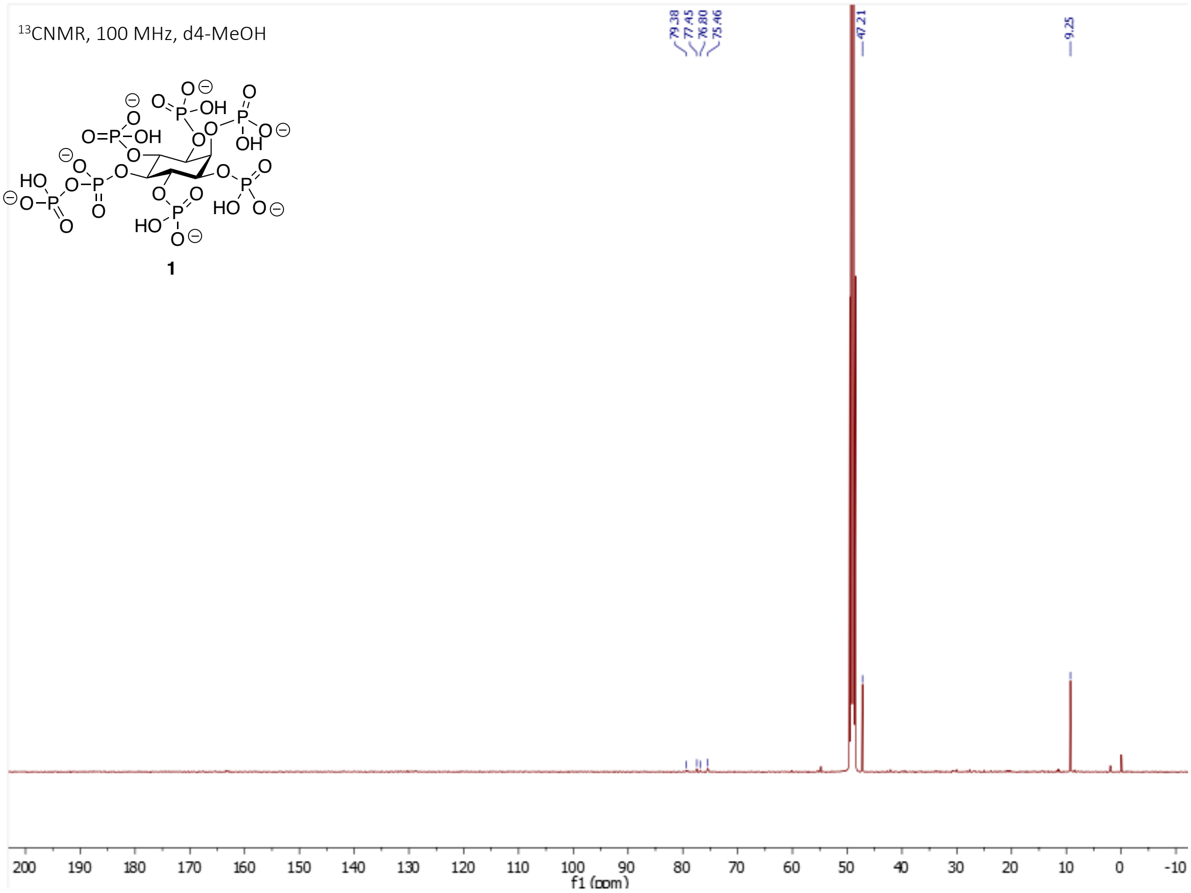
13CNMR, 100 MHz, d4-MeOH
1
f1 (ppm)

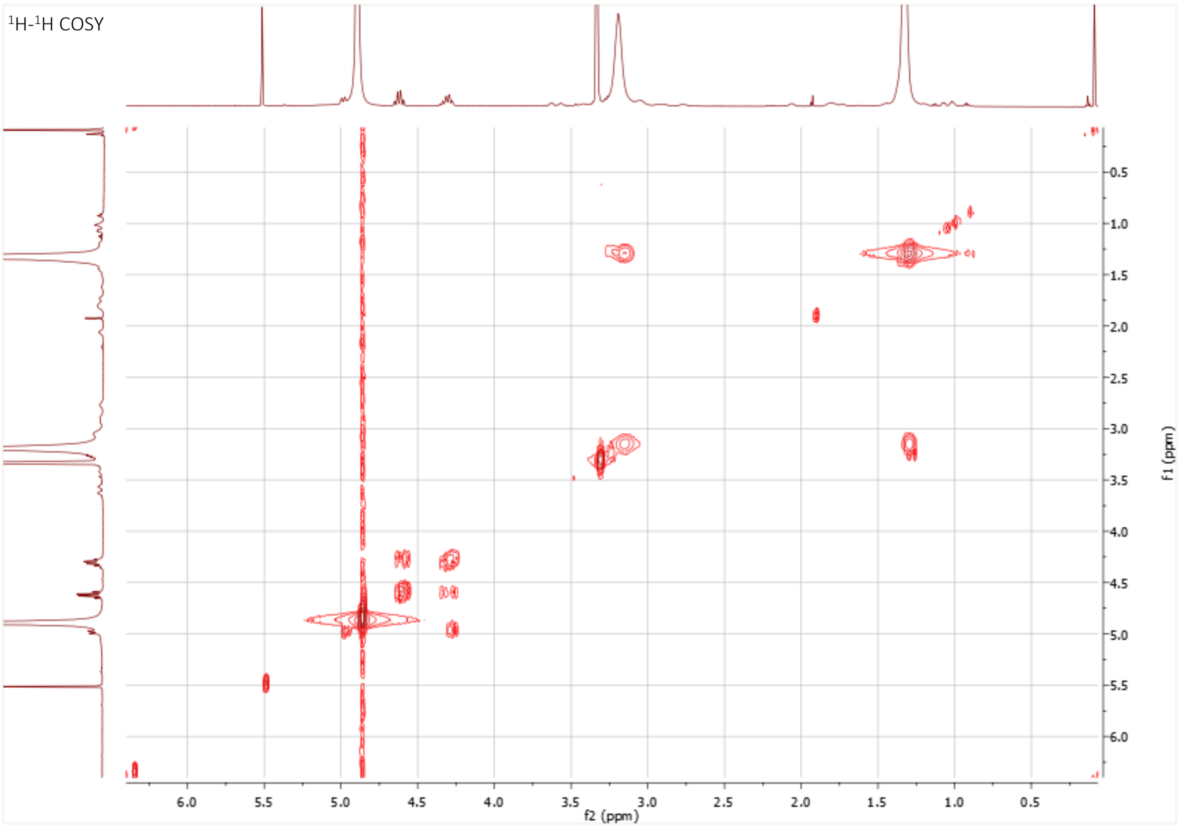

^{1}H-^{1}H COSY
f2 (ppm)
f1 (ppm)

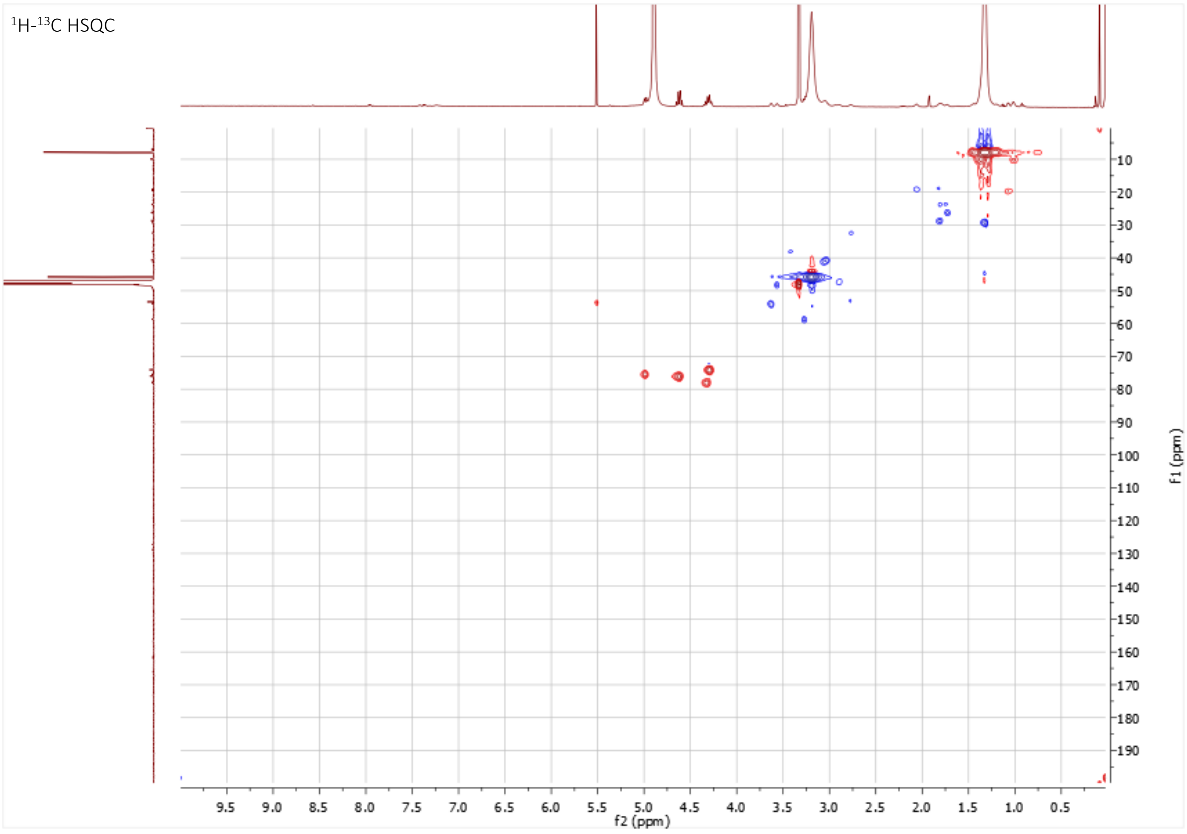

^{1}H-^{13}C HSQC
f2 (ppm)
f1 (ppm)

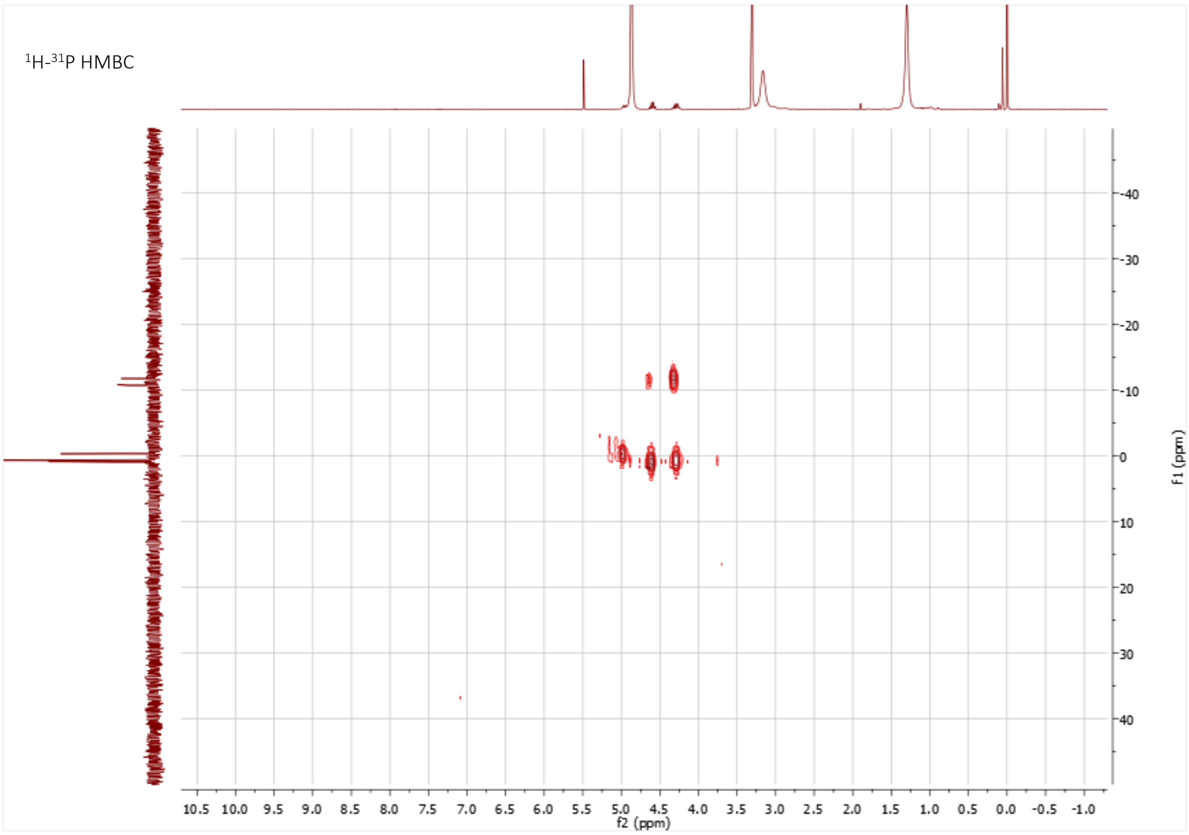

^{1}H-^{31}P HMBC
f2 (ppm)
f1 (ppm)

Photophysical measurements of [Eu.3]+

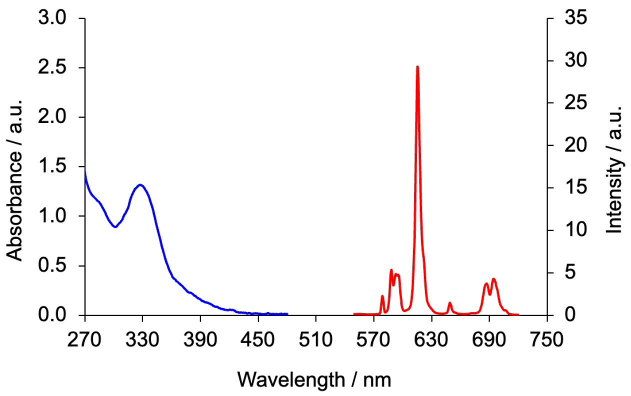

Figure S1. Large separation between the absorption and emission spectrum of [**Eu.3**]+ (5 μM), measured in 10 mM HEPES at pH 7.0 and 295 K.

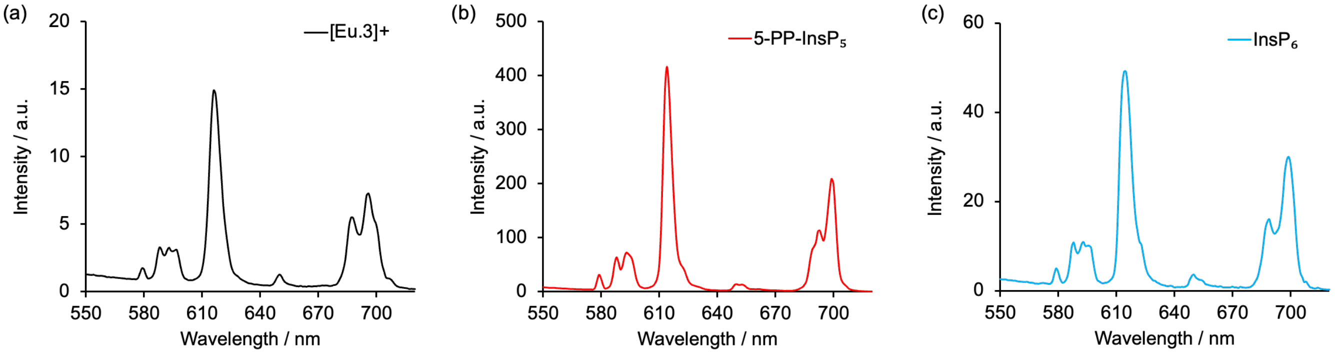

Figure S2. Emission spectral form of [**Eu.3**]+ (5 μM) (a) alone, (b) with added 5-PP-InsP5 (250 μM), (c) with added Insp6 (250 μM). Measured in 10 mM HEPES at pH 7.0 and 295 K, λ_{exc} = 330 nm.

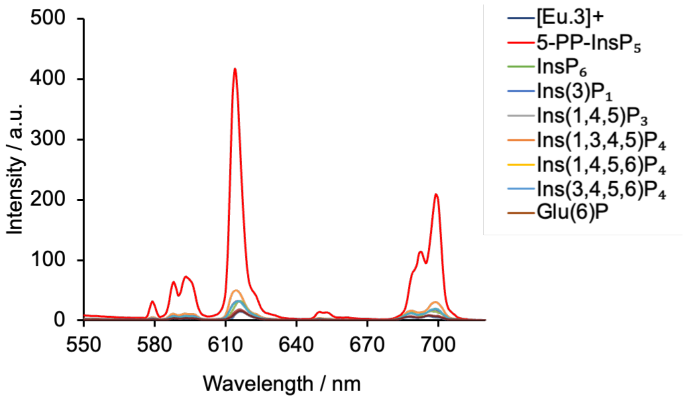

Figure S3. Large enhancement in emission intensity of [**Eu.3**]+ (5 μM) with added 5-PP-InsP5 (250 μM) compared with the minor emission changes with added inositol phosphates including InsP6, Ins(3)P1, Ins(1,4,5)P3, Ins(1,3,4,5)P4, Ins(1,3,5,56)P4, Ins(3,4,5,6)P4, and glucose-6-phosphate (250 μM each). Measured in 10 mM HEPES at pH 7.0 and 295 K, λ_{exc} = 330 nm.

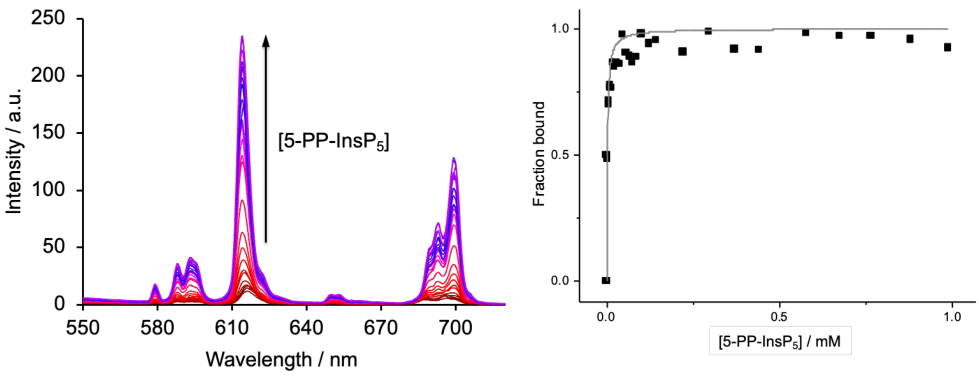

Figure S4. Binding titration of **[Eu.3]**$^+$ with 5-PP-InsP$_5$. (a) Change in emission spectrum upon incremental addition of PP-InsP$_5$. (b) Plot of fraction bound (determined from $\Delta J = 2$ / $\Delta J = 1$ intensity ratio) versus concentration of 5-PP-InsP$_5$, showing the fit to a 1:1 binding isotherm. Data shown are a representative example of two independent experiments. Conditions: 5 μM **[Eu.3]**$^+$ in 10 mM HEPES, pH 7.0, 295 K, λ_{ex} = 330 nm.

Data obtained from fitting of the titration of [Eu.3]$^+$ with 5-PP-InsP$_5$ to a 1:1 and 2:1 binding model using Bindfit, can be accessed here:

1:1 host-guest model:

http://app.supramolecular.org/bindfit/view/96b69857-dd92-4bb8-89f5-75a8cfbc9335

2:1 host-guest model:

http://app.supramolecular.org/bindfit/view/54645a73-4e6b-4c99-a44e-f9b76ded4025

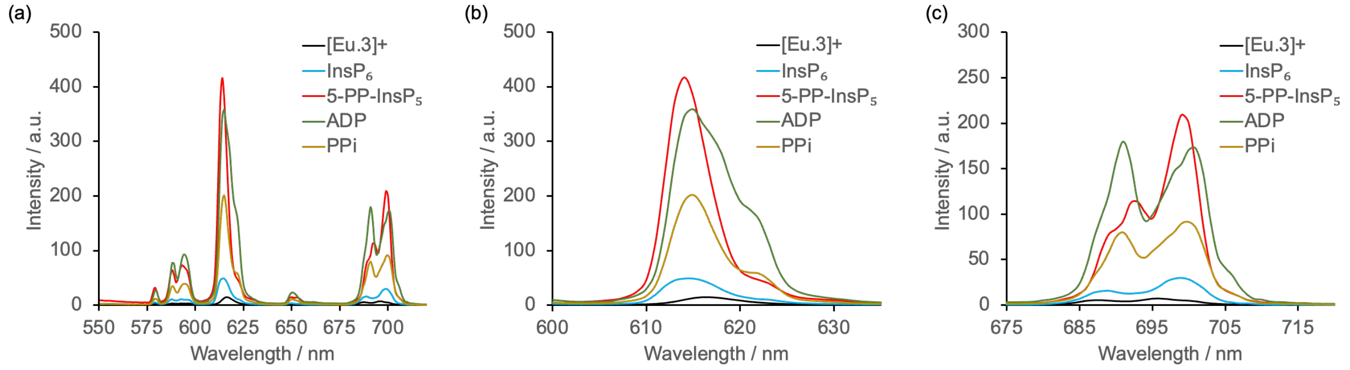

Figure S5. Comparison of emission spectral changes of [**Eu.3**]$^+$ (5 μM) with added 5-PP-InsP$_5$ (red line), InsP$_6$ (light blue), ADP (green) and pyrophosphate (purple) (250 μM each). (a) Overall Eu emission, (b) Expansion of the $\Delta J = 2$ band (600 – 630 nm) and (c) $\Delta J = 4$ band (675 – 715 nm). Measured in 10 mM HEPES at pH 7.0 and 295 K, λ_{exc} = 330 nm.

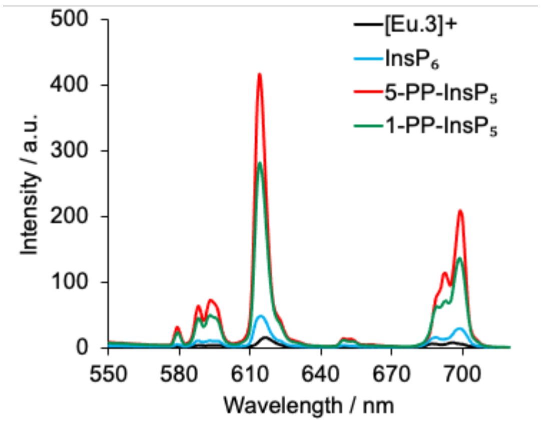

Figure S6. Comparison of emission spectral changes of [**Eu.3**]$^+$ (5 μM) with added 5-PP-InsP$_5$ (red line), 1-PP-InsP$_5$ (green) and InsP$_6$ (light blue) (250 μM each). Measured in 10 mM HEPES at pH 7.0 and 295 K, λ_{exc} = 330 nm.

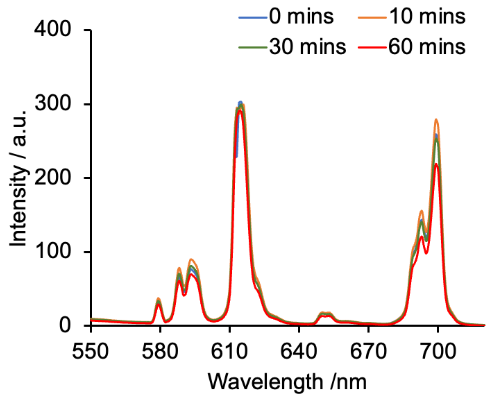

Figure S7. Minimal variation in emission spectra of [**Eu.3**]$^+$ (5 μM) in the presence of 5-PP-InsP$_5$ (250 μM) over a 60 minute incubation period. Measured in 10 mM HEPES at pH 7.0 and 295 K, λ_{exc} = 330 nm.

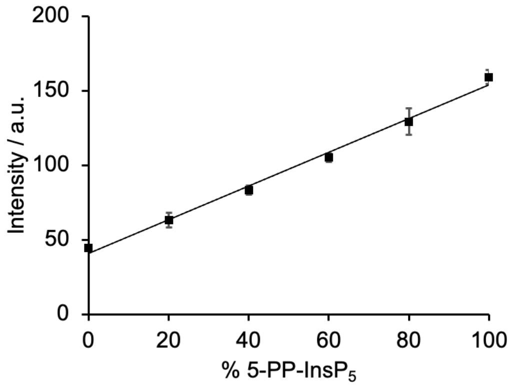

Figure S8. Enzyme simulation reaction demonstrating a linear increase in time-resolved emission of **[Eu.3]**$^+$ with increasing 5-PP-InsP$_5$/ InsP$_6$ molar ratio in the presence of bovine serum albumin (BSA). Data are mean ± SEM ($r^2 > 0.99$) from experiments performed using 5 μM **[Eu.3]**$^+$, 0.2 mg/mL BSA , 100 μM total 5-PP-InsP$_5$ + InsP$_6$, in 10 mM HEPES, pH 7.0.

DFT-optimised molecular structures and *ab initio* luminescence spectra

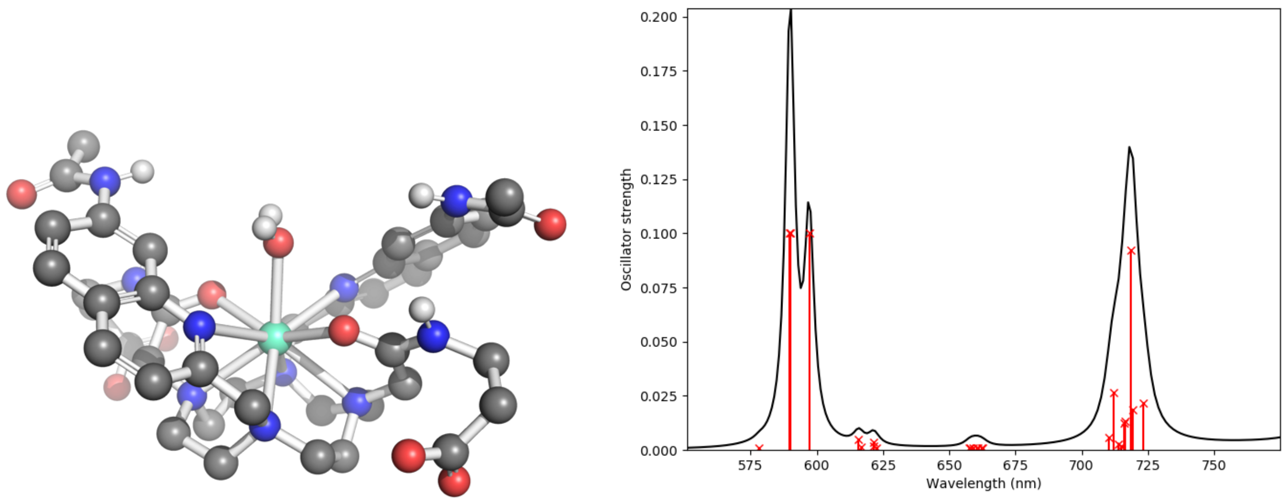

Figure S9. 3D-Molecular structure and simulated luminescence spectrum of the water-bound complex.

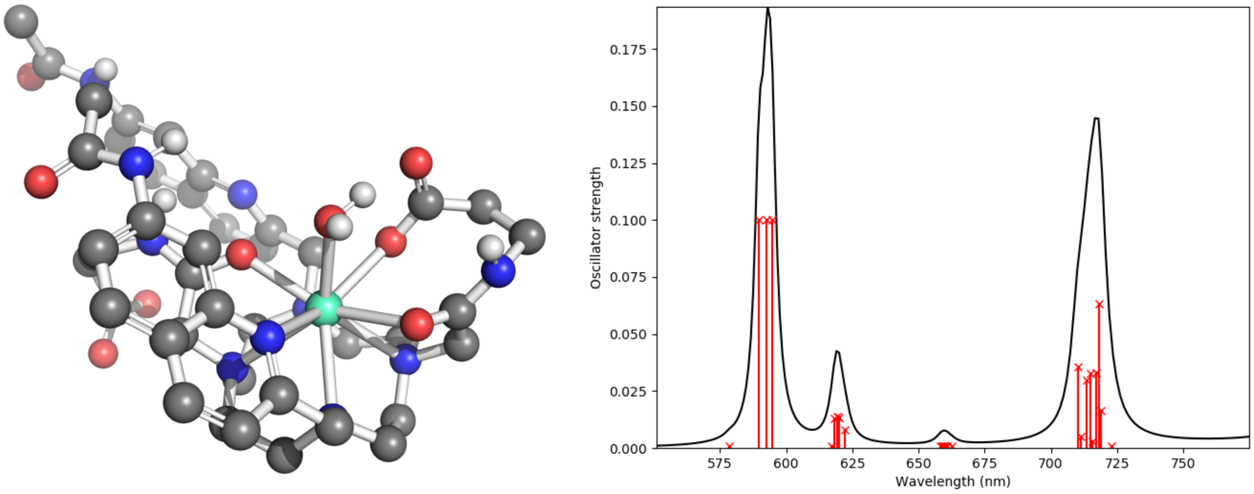

Figure S10. 3D-Molecular structure and simulated luminescence spectrum of an alternative conformer of the water-bound complex with the quinoline arm exchanged for a carboxylic acid group. This structure was found to be higher in free energy than the above one by 17.0 kJ/mol.

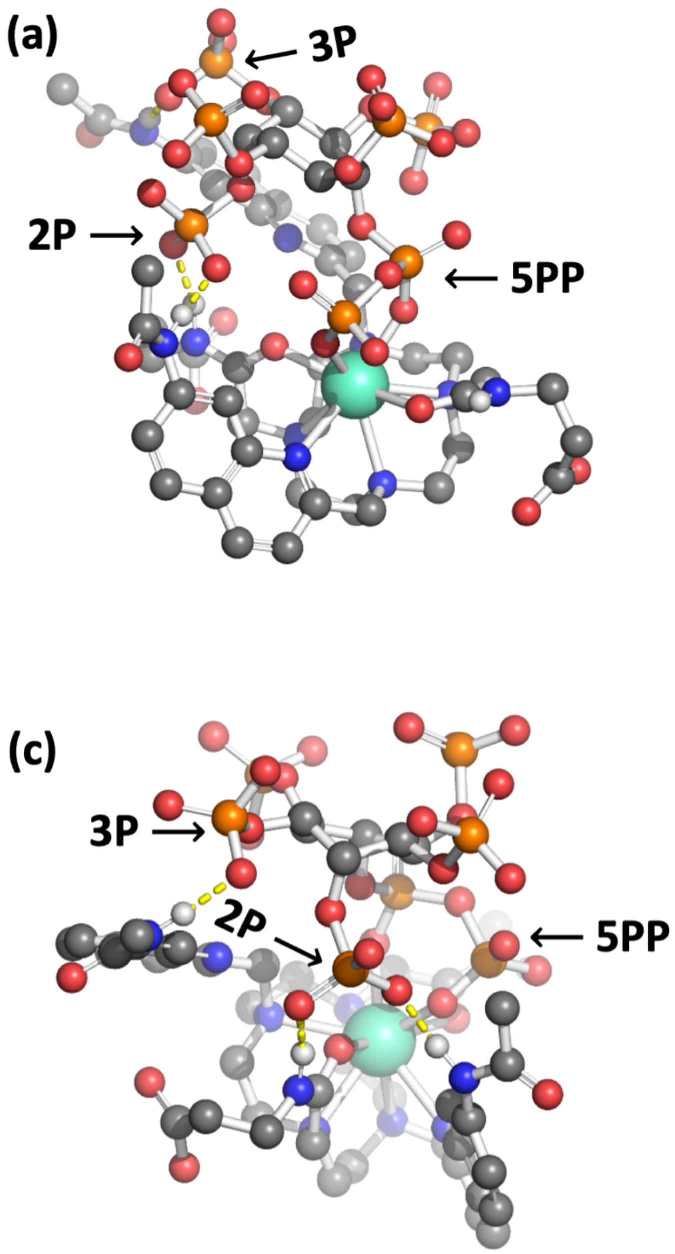

Figure S11. Alternative depictions of the 5-PP-InsP_5 bound structure, highlighting the hydrogen bonds formed (as yellow dashed lines) and the dissociation of one of the quinoline arms (panel (a), background). The phosphate groups involved in hydrogen bonding (2P, 3P) and the pyrophosphate group (5PP) are labelled for clarity.

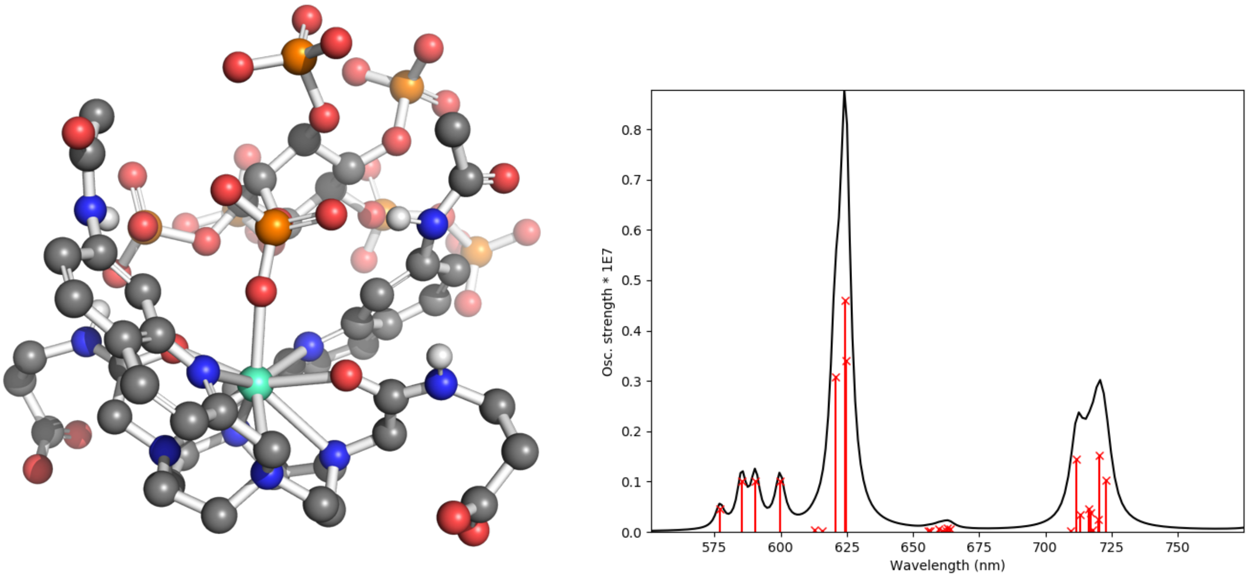

Figure S12. Monodentate binding mode of 5-PP-InsP$_5$. This structure was found to be 9.2 kJ/mol higher in energy than the bidentate binding mode.

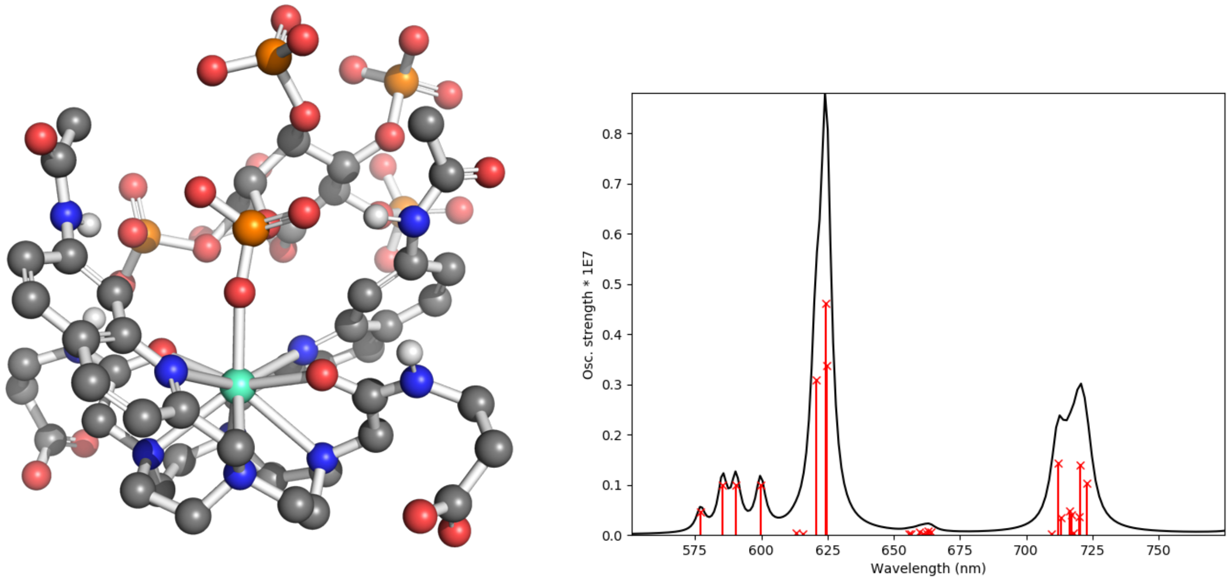

Figure S13. Most stable binding mode of InsP$_6$ (featuring binding in axial position). This structure showed reduced binding by 22.3 kJ/mol compared to the bidentate binding mode of 5-PP-InsP$_5$.

References

1. D. Lampe, C. Liu and B. V. L. Potter, *J. Med. Chem.*, 1994, **37**, 907-912.
2. A. Beeby, I. M. Clarkson, R. S. Dickins, S. Faulkner, D. Parker, L. Royle, A. S. de Sousa, J. A. Gareth Williams and M. Woods, *J. Chem. Soc., Perkin Trans. 2*, 1999, **0**, 493-504.
3. P. C. Hariharan and J. A. Pople, *Theor. Chim. Acta*, 1973, **28**, 213-222.
4. N. Mardirossian and M. Head-Gordon, *J. Chem. Phys.*, 2016, **144**, 214110.
5. M. Dolg, H. Stoll and H. Preuss, *J. Chem. Phys.*, 1989, **90**, 1730-1734.
6. A. V. Marenich, C. J. Cramer and D. G. Truhlar, *J. Phys. Chem. B*, 2009, **113**, 6378-6396.
7. E. Epifanovsky, A. T. B. Gilbert, X. Feng, J. Lee, Y. Mao, N. Mardirossian, P. Pokhilko, A. F. White, M. P. Coons and A. L. Dempwolff, *J. Chem. Phys.*, 2021, **155**, 084801.
8. F. Neese, *WIREs Comput. Mol. Sci.*, 2022, **12**, e1606.
9. F. Weigend and R. Ahlrichs, *Phys.Chem. Chem. Phys.*, 2005, **7**, 3297-3305.
10. H. Kruse and S. Grimme, *J. Chem. Phys.*, 2012, **136**, 04B613.
11. B. O. Roos, R. Lindh, P.-Å. Malmqvist, V. Veryazov and P.-O. Widmark, *J. Phys. Chem. A*, 2004, **108**, 2851-2858.
12. P.-Å. Malmqvist and B. O. Roos, *Chem. Phys. Lett.*, 1989, **155**, 189-194.
13. I. Fdez. Galván, M. Vacher, A. Alavi, C. Angeli, F. Aquilante, J. Autschbach, J. J. Bao, S. I. Bokarev, N. A. Bogdanov, R. K. Carlson, L. F. Chibotaru, J. Creutzberg, N. Dattani, M. G. Delcey, S. S. Dong, A. Dreuw, L. Freitag, L. M. Frutos, L. Gagliardi, F. Gendron, A. Giussani, L. González, G. Grell, M. Guo, C. E. Hoyer, M. Johansson, S. Keller, S. Knecht, G. Kovačević, E. Källman, G. Li Manni, M. Lundberg, Y. Ma, S. Mai, J. P. Malhado, P. Å. Malmqvist, P. Marquetand, S. A. Mewes, J. Norell, M. Olivucci, M. Oppel, Q. M. Phung, K. Pierloot, F. Plasser, M. Reiher, A. M. Sand, I. Schapiro, P. Sharma, C. J. Stein, L. K. Sørensen, D. G. Truhlar, M. Ugandi, L. Ungur, A. Valentini, S. Vancoillie, V. Veryazov, O. Weser, T. A. Wesołowski, P.-O. Widmark, S. Wouters, A. Zech, J. P. Zobel and R. Lindh, *J. Chem. Theory Comput.*, 2019, **15**, 5925-5964.
14. H. Wang, H. Y. Godage, A. M. Riley, J. D. Weaver, S. B. Shears and B. V. L. Potter, *Chem. Biol.*, 2014, **21**, 689-699.
15. A. M. Riley, H. Wang, J. D. Weaver, S. B. Shears and B. V. L. Potter, *Chem. Commun.*, 2012, **48**, 11292-11294.
16. S. B. Engelsma, N. J. Meeuwenoord, H. S. Overkleeft, G. A. van der Marel and D. V. Filippov, *Angew. Chem. Int. Ed.*, 2017, **56**, 2955-2959.
17. A. M. Riley, J. E. Unterlass, V. Konieczny, C. W. Taylor, T. Helleday and B. V. L. Potter, *MedChemComm*, 2018, **9**, 1105-1113.
